# A synthesis of evidence for policy from behavioural science during COVID-19

**DOI:** 10.1038/s41586-023-06840-9

**Published:** 2023-12-13

**Authors:** Kai Ruggeri, Friederike Stock, S. Alexander Haslam, Valerio Capraro, Paulo Boggio, Naomi Ellemers, Aleksandra Cichocka, Karen M. Douglas, David G. Rand, Sander van der Linden, Mina Cikara, Eli J. Finkel, James N. Druckman, Michael J. A. Wohl, Richard E. Petty, Joshua A. Tucker, Azim Shariff, Michele Gelfand, Dominic Packer, Jolanda Jetten, Paul A. M. Van Lange, Gordon Pennycook, Ellen Peters, Katherine Baicker, Alia Crum, Kim A. Weeden, Lucy Napper, Nassim Tabri, Jamil Zaki, Linda Skitka, Shinobu Kitayama, Dean Mobbs, Cass R. Sunstein, Sarah Ashcroft-Jones, Anna Louise Todsen, Ali Hajian, Sanne Verra, Vanessa Buehler, Maja Friedemann, Marlene Hecht, Rayyan S. Mobarak, Ralitsa Karakasheva, Markus R. Tünte, Siu Kit Yeung, R. Shayna Rosenbaum, Žan Lep, Yuki Yamada, Sa-kiera Tiarra Jolynn Hudson, Lucía Macchia, Irina Soboleva, Eugen Dimant, Sandra J. Geiger, Hannes Jarke, Tobias Wingen, Jana B. Berkessel, Silvana Mareva, Lucy McGill, Francesca Papa, Bojana Većkalov, Zeina Afif, Eike K. Buabang, Marna Landman, Felice Tavera, Jack L. Andrews, Aslı Bursalıoğlu, Zorana Zupan, Lisa Wagner, Joaquín Navajas, Marek Vranka, David Kasdan, Patricia Chen, Kathleen R. Hudson, Lindsay M. Novak, Paul Teas, Nikolay R. Rachev, Matteo M. Galizzi, Katherine L. Milkman, Marija Petrović, Jay J. Van Bavel, Robb Willer

**Affiliations:** 1 Department of Health Policy and Management, Columbia University Mailman School of Public Health, New York City, NY USA; 2 Policy Research Group, Centre for Business Research, Judge Business School, University of Cambridge, Cambridge, UK; 3 274th ASOS, US Air Force/New York Air National Guard, Syracuse, NY United States; 4 Center for Adaptive Rationality, Max Planck Institute for Human Development, Berlin, Germany; 5 Department of Psychology, Humboldt University of Berlin, Berlin, Germany; 6 University of Queensland, St Lucia, Queensland Australia; 7 University of Milan-Bicocca, Milan, Italy; 8 Mackenzie Presbyterian University, São Paulo, Brazil; 9 National Institute of Science and Technology on Social and Affective Neuroscience, CNPq, São Paulo, Brazil; 10 Utrecht University, Utrecht, Netherlands; 11 University of Kent, Canterbury, UK; 12 Massachusetts Institute of Technology, Cambridge, MA USA; 13 Department of Psychology, University of Cambridge, Cambridge, UK; 14 Harvard University, Cambridge, MA USA; 15 Department of Psychology, Northwestern University, Evanston, IL USA; 16 Kellogg School of Management, Northwestern University, Evanston, IL USA; 17 Northwestern University, Evanston, IL USA; 18 Department of Psychology, Carleton University, Ottawa, Ontario Canada; 19 Department of Psychology, Ohio State University, Columbus, OH USA; 20 Department of Politics & Center for Social Media and Politics, New York University, New York, NY USA; 21 Department of Psychology, University of British Columbia, Vancouver, British Columbia Canada; 22 Stanford University, Stanford, CA USA; 23 Lehigh University, Bethlehem, PA USA; 24 Institute for Brain and Behavior Amsterdam, Department of Experimental and Applied Psychology, Vrije Universiteit Amsterdam, Amsterdam, Netherlands; 25 Global Faculty, Social and Economic Behavior, University of Cologne, Cologne, Germany; 26 Cornell University, Ithaca, NY USA; 27 Center for Science Communication Research, School of Journalism and Communication, University of Oregon, Eugene, OR USA; 28 Psychology Department, University of Oregon, Eugene, OR USA; 29 University of Chicago, Chicago, IL USA; 30 Department of Psychology, Stanford University, Stanford, CA USA; 31 University of Illinois Chicago, Chicago, IL USA; 32 University of Michigan, Ann Arbor, MI USA; 33 Department of Humanities and Social Sciences, California Institute of Technology, Pasadena, CA USA; 34 Computation and Neural Systems Program, California Institute of Technology, Pasadena, CA USA; 35 Department of Experimental Psychology, University of Oxford, Oxford, UK; 36 Department of Social Policy and Evaluation, University of Oxford, Oxford, UK; 37 University of Tehran, Tehran, Iran; 38 Cowry Consulting, London, UK; 39 University of Oxford, Oxford, UK; 40 Department of Agricultural and Resource Economics, University of Maryland, College Park, MD USA; 41 Junior Researcher Programme, Cambridge, UK; 42 Department of Developmental and Educational Psychology, Faculty of Psychology, University of Vienna, Vienna, Austria; 43 Department of Psychology, The Chinese University of Hong Kong, Hong Kong SAR, China; 44 Department of Psychology, York University, Toronto, Ontario Canada; 45 Rotman Research Institute, Baycrest Academy for Research and Education, Toronto, Ontario Canada; 46 Department of Psychology, Faculty of Arts, University of Ljubljana, Ljubljana, Slovenia; 47 Centre for Applied Epistemology, Educational Research Institute, Ljubljana, Slovenia; 48 Faculty of Arts and Science, Kyushu University, Fukuoka, Japan; 49 Haas School of Business, University of California Berkeley, Berkeley, CA USA; 50 City, University of London, London, UK; 51 Duke Kunshan University, Kunshan, China; 52 Center for Social Norms and Behavioral Dynamics, University of Pennsylvania, Philadelphia, PA USA; 53 CESifo, Munich, Germany; 54 Environmental Psychology, Department of Cognition, Emotion, and Methods in Psychology, Faculty of Psychology, University of Vienna, Vienna, Austria; 55 University of Bonn, University Hospital Bonn, Institute of General Practice and Family Medicine, Bonn, Germany; 56 Mannheim Centre for European Social Research, University of Mannheim, Mannheim, Germany; 57 MRC Cognition and Brain Sciences Unit, University of Cambridge, Cambridge, UK; 58 Psychology Department, Faculty of Health and Life Sciences, University of Exeter, Exeter, UK; 59 University College Dublin, Dublin, Ireland; 60 University of Groningen, Groningen, Netherlands; 61 Organisation for Economic Co-operation and Development, Paris, France; 62 University of Amsterdam, Amsterdam, Netherlands; 63 The World Bank, Washington DC, USA; 64 Trinity College Institute of Neuroscience, Trinity College Dublin, Dublin, Ireland; 65 Gordon Institute of Business Science, University of Pretoria, Johannesburg, South Africa; 66 Department of Psychology, University of Cologne, Cologne, Germany; 67 University College, Oxford, UK; 68 Department of Psychology, Loyola University Chicago, Chicago, IL USA; 69 Institute of Psychology, Faculty of Philosophy, University of Belgrade, Belgrade, Serbia; 70 Jacobs Center for Productive Youth Development, University of Zurich, Zurich, Switzerland; 71 Department of Psychology, University of Zurich, Zurich, Switzerland; 72 Laboratorio de Neurociencia, Universidad Torcuato Di Tella, Buenos Aires, Argentina; 73 Escuela de Negocios, Universidad Torcuato Di Tella, Buenos Aires, Argentina; 74 Consejo Nacional de Investigaciones Científicas y Técnicas (CONICET), Buenos Aires, Argentina; 75 Charles University, Prague, Czech Republic; 76 Sungkyunkwan University, Seoul, Republic of Korea; 77 University of Texas at Austin, Austin, TX USA; 78 National University of Singapore, Singapore, Singapore; 79 Department of General, Experimental, Developmental, and Health Psychology, Sofia University St. Kliment Ohridski, Sofia, Bulgaria; 80 Department of Psychological and Behavioural Science, London School of Economics, London, UK; 81 The Wharton School of the University of Pennsylvania, Philadelphia, PA USA; 82 Department of Psychology & Laboratory for Research of Individual Differences, Faculty of Philosophy, University of Belgrade, Belgrade, Serbia; 83 Department of Psychology & Center for Neural Science, New York University, New York, NY USA; 84 Department of Sociology, Stanford University, Stanford, CA USA

**Keywords:** Human behaviour, Society, Policy, Psychology

## Abstract

Scientific evidence regularly guides policy decisions^1^, with behavioural science increasingly part of this process^2^. In April 2020, an influential paper^3^ proposed 19 policy recommendations (‘claims’) detailing how evidence from behavioural science could contribute to efforts to reduce impacts and end the COVID-19 pandemic. Here we assess 747 pandemic-related research articles that empirically investigated those claims. We report the scale of evidence and whether evidence supports them to indicate applicability for policymaking. Two independent teams, involving 72 reviewers, found evidence for 18 of 19 claims, with both teams finding evidence supporting 16 (89%) of those 18 claims. The strongest evidence supported claims that anticipated culture, polarization and misinformation would be associated with policy effectiveness. Claims suggesting trusted leaders and positive social norms increased adherence to behavioural interventions also had strong empirical support, as did appealing to social consensus or bipartisan agreement. Targeted language in messaging yielded mixed effects and there were no effects for highlighting individual benefits or protecting others. No available evidence existed to assess any distinct differences in effects between using the terms ‘physical distancing’ and ‘social distancing’. Analysis of 463 papers containing data showed generally large samples; 418 involved human participants with a mean of 16,848 (median of 1,699). That statistical power underscored improved suitability of behavioural science research for informing policy decisions. Furthermore, by implementing a standardized approach to evidence selection and synthesis, we amplify broader implications for advancing scientific evidence in policy formulation and prioritization.

## Abstract (web summary)

Evaluation of evidence generated to test 19 proposed policy recommendations and guidance for the future.

## Main

Scientific evidence has an important role in policy decisions^1^. This has been increasingly true of evidence from the behavioural and social sciences^2^, particularly for public health policy throughout the global COVID-19 pandemic^4,5^. One broad challenge in this process is that there is no universally endorsed approach to determine which scientific insights should inform policy^6^. Recommendations may be made on an ad hoc basis, may be based on relationships between certain researchers and policymakers, and may fail to factor in an appropriate level of uncertainty^7–9^. This is further complicated by the sheer volume and heterogeneity of evidence, making appropriate identification and synthesis a substantial challenge^10^.

One major example of science impacting policy comes from April 2020, when 42 academics from 8 countries and multiple academic disciplines published a review containing a series of hypotheses about factors that were likely to shape collective behaviour during a pandemic^3^. Topics included threat and risk perception, social norms, science communication, emphasizing the importance of individual and collective interests, leadership, stress and coping. The paper also included a list of broad behavioural insights (which we refer to as ‘claims’) deemed most relevant to the pandemic. The article received unprecedented attention: in only 2 years, it was cited over 3,000 times, and by December 2022 had an Altmetric score in the highest 0.0001% of all articles ever published. This was, in part, because governments around the world formulated pandemic policy strategies explicitly^11^ on the basis of the behavioural concepts highlighted in the paper^12–18^. However, it was also because social and behavioural scientists viewed the pandemic as a critical focus for their attention^19^ and research^20,21^.

Naturally, with such levels of visibility, concerns were raised about various claims made by Van Bavel et al.^3^. This led to some concerns about academics making recommendations despite not all having previous experience in the domain of public policy or public health^22,23^. In the context of the so-called replication crisis in psychology, it has also been suggested that the article focused too much on evidence from WEIRD (Western, educated, industrialized, rich, democratic) populations, took insufficient account of the heterogeneity of effects, overstated the validity of existing evidence and was opportunistic^24–27^.

This paper responds to those concerns by retrospectively evaluating the quality of the claims by Van Bavel et al.^3^. Although, more broadly, it presents a valuable opportunity to mobilize many independent experts using a structured approach to assess the appropriateness of scientific evidence being considered for application to policy. This is important not only to inform theorizing about behaviour during a pandemic but also to establish the relevance of these claims for future emergencies and more generally across public policy. It is an equally important complement to addressing replicability in science, by scrutinizing evidence produced during a crisis while also revisiting previous claims made by experts.

Concerns related to the readiness or robustness of evidence for application to policy are neither new nor unique to COVID-19. Terms such as ‘evidence-based policy’ have long been used to apply research to major decisions in government, institutions, schools and businesses^28^. The lack of consensus concerning what counts as sufficient evidence for policy decisions^29^ is a particular problem in an emergency when policymakers must take urgent actions with sometimes limited evidence. As such, they may seek the lowest-risk or most-effective approach rather than a perfectly informed approach (which is often unavailable or unclear)^30^, or they may risk delays that create even greater harm^31,32^. Consequently, the COVID-19 pandemic presented an opportunity to assess evidence in a way that encouraged more evaluations of academic policy recommendations in the future.

With such a substantial amount of evidence now available, our goal was to provide a descriptive synthesis of evidence available relevant to 19 claims from the Van Bavel et al. article (see the first column in Table 1 from this paper for the full statements). In essence, we evaluated the extent to which those statements provided valid policy guidance, privileging empirical evidence of consistent real-world impact. The present work is not a comprehensive evaluation of all behavioural science related to the pandemic, which would be beyond the scope of a single article, but only of the key statements made in the 2020 paper.

**Table 1:** Nineteen claims about social and behavioural science during the COVID-19 pandemic

2020 Claim wording	Revised claim wording	Behavioural theme
(1) A shared sense of identity or purpose can be encouraged by addressing the public in collective terms and by urging ‘us’ to act for the common good	There is a small positive association between collective identity and behaviour for the common good, but the relationship depends on the level of identity activated (for example, nation versus European Union)	Sense of identity
(2) Identifying trusted sources (for example, local, religious or community leaders) that are credible to different audiences to share public health messages can be effective	Identifying trusted sources (for example, local, religious, political or community leaders) that are credible to different audiences to share public health messages can be effective in increasing intentions to engage in recommended health behaviours	Trust and leadership
(3) Leaders and the media might try to promote cooperative behaviour by emphasizing that cooperating is the right thing to do and that other people are already cooperating	Emphasizing cooperation and highlighting the cooperative behaviour of other people can encourage people to adhere to public health recommendations, although effects may be small	Trust and leadership
(4) Norms of prosocial behaviour are more effective when coupled with the expectation of social approval and modelled by in-group members who are central in social networks	Surveys have shown that descriptive norms, especially when enacted by close reference groups, are associated with greater compliance with public health recommendations and self-reported prosocial behaviours	Sense of identity
(5) Leaders and members of the media should highlight bipartisan support for COVID-related measures, when they exist, as such endorsements in other contexts have reduced polarization and led to less-biased reasoning	Where polarization regarding public health behaviours exists, endorsement from bipartisan coalitions can be effective in reducing polarization and increasing compliance	Messaging and language
(6) There is a need for more targeted public health information within marginalized communities and for partnerships between public health authorities and trusted organizations that are internal to these communities	Marginalized communities have very different risks and health outcomes and may receive different information through different channels, suggesting the potential benefit of targeted communication and strategies	Messaging and language
(7) Messages that (i) emphasize benefits to the recipient, (ii) focus on protecting others, (iii) align with the moral values of recipients, (iv) appeal to social consensus or scientific norms, and/or (v) highlight the prospect of social group approval tend to be persuasive	Messages may be more effective when they align closely with the moral values of recipients, appeal to social consensus or scientific norms, and highlight group approval	Messaging and language
(8) Given the importance of slowing infections, it may be helpful to make people aware that they benefit from others’ access to preventative measures	There is suggestive, albeit little, empirical evidence that it can help to make people aware that they benefit from others’ access to preventative measures	Sense of identity
(9) Preparing people for misinformation and ensuring they have accurate information and counterarguments against false information before they encounter conspiracy theories, fake news or other forms of misinformation can help to inoculate them against false information	Preparing people for misinformation before they encounter conspiracy theories, fake news or other forms of misinformation — for example, by ensuring that they have accurate information and counterarguments against false information, or by prompting them to consider accuracy — can help to reduce belief in, and/or sharing of, false information for a limited time	Social cohesion and misinformation
(10) Use of the term ‘social distancing’ might imply that one needs to cut off meaningful interactions. A preferable term is ‘physical distancing’, because it allows for the fact that social connection is possible even when people are physically separated	Although ‘physical distancing’ is a more accurate term than ‘social distancing’ and may encourage more social connection, there is no evidence on whether it is more effective in encouraging public health behaviours	Messaging and language
(11) As negative emotions increase, people may rely on negative information about COVID-19 more than other information to make decisions. In the case of strong emotional reactions, people may also ignore important numeric information such as probabilities and the scope of a problem	An increase in negative emotions related to the pandemic may influence behaviour and decision-making and lead people to ignore important information, such as probabilities of negative outcomes or actual risk level	Messaging and language
(12) Cultures accustomed to prioritizing freedom over security may also have more difficulty coordinating in the face of a pandemic	Strong correlations indicate that cultures accustomed to prioritizing freedom over security may also have more difficulty coordinating in the face of a pandemic	Social cohesion and misinformation
(13) Fake news, conspiracy theories and misinformation will have a negative effect on vaccine hesitancy	Evidence has shown that fake news, conspiracy theories and misinformation were negatively associated with vaccination intentions, but the effect on actual vaccination behaviour has not been shown	Social cohesion and misinformation
(14) Unmitigated political polarization will disrupt or create other negative effects on attempts to minimize or end the pandemic	Evidence has shown that divergent partisan identities lead to significantly different opinions and reported behaviours in response to the pandemic, undermining coordination efforts to minimize or end the pandemic	Social cohesion and misinformation
(15) Active use of online connections can reduce some negative mental and other health effects created by isolation policies	Active social connections online can buffer against negative mental health effects, although mitigating effects may be small	Sense of identity

Claims in the left column show the original wording from Van Bavel et al.^3^. The text in the right column shows updated wording after assessing the evidence in 2022. Note that claim 7 has five components, making a total of 19 for the table.

We assessed 19 behavioural policy recommendations through evaluating available articles based on the level of evidence they include. Ratings range from purely opinion or theory to large-scale, replicated field studies, as well as the size and direction of effects reported (see ‘Procedure’ in the Methods section). Our evaluations primarily focused on the scale and scope of empirical findings directly related to the claims, although the compiled data (available at https://psyarxiv.com/58udn) also highlights methods, geographical settings and specific behaviours. We then synthesized the evidence within each claim to formulate a summary evaluation. For this exercise, we included both original authors and an independent team of evaluators to select and assess evidence relevant to these claims (see ‘Evaluation teams’ in the Methods section), all of whom were blinded to names and assessments. This allowed us to leverage the expertise of the original authors while also adding a diverse group of scholars who were not involved in the original paper to provide an independent, objective evaluation of the evidence.

Our primary motivations are to (1) transparently evaluate ex post evidence for a set of highly influential claims regarding behaviour during a pandemic, (2) implement a pragmatic, expert-driven method for evaluating and synthesizing evidence that is suitable for informing public policy (both related to COVID-19 and future applications), and (3) make those assessments public in a way that promotes transparency and builds trust with the public^33^. The first and second aims are broadly relevant across all scientific research. The third aim is specifically relevant to assessing policy recommendations, which is why we decided to provide a descriptive summary rather than focus on methods or causal inference (more highly valued in science). The first aim is also especially critical given substantial concerns about public trust in science in general^34^ and raised directly in the context of COVID-19 (refs. ^35–39^).

Evaluating predictions of academic experts is an important exercise to protect against questionable research practices, mistakes and overconfidence^38,40,41^, which were a common concern specifically in behavioural science during COVID-19. Those concerns fed into cautions raised about systematic reviews of available evidence^42,43^. Furthermore, by mobilizing a large group of fully independent reviewers, we ensure that no single paper (whether highly powered or merely highly visible) or person can have unchecked influence on the evaluation of policy-relevant evidence.

## Evidence for 19 pandemic behaviour claims

**Extended Data Fig. 1:**
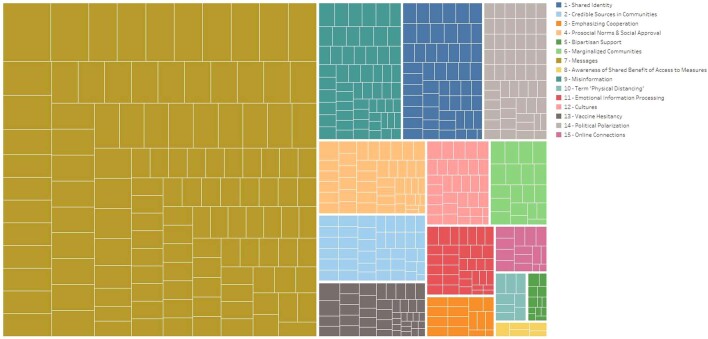
Breakdown of articles reviewed by claim. Each box in the figure represents an article reviewed, with the color indicating which claim it fit under. For this figure, all sections within claim 7 are treated as a single claim due to most papers in that claim covering multiple sections. The size of each box reflects how many reviewers gave the article a rating. Specific claim colors are indicated in the legend.

As outlined in the Methods (‘Procedure and evidence used for evaluations’), all 747 articles were reviewed by at least two reviewers from each team (four total); 518 articles received at least one rating (see Extended Data Fig. 1). One-hundred and eighty-six articles were unanimously rated as not directly relevant or informative to the claim; 43 were found to be duplicated work, typically papers that had changed titles from preprint to publication. Of the 19 claims, 18 had at least some empirical evidence to assess (see full descriptions in Tables 2–6), with only one claim lacking any empirical research. Of those 18 claims, 13 claims were assessed as having been studied empirically, although only in surveys or limited laboratory settings (see Extended Data Table 1 for a breakdown of articles).

**Table 2:** Evidence assessments for four claims on sense of identity

Claim (number)	Evidence	Level	Direction	Effect size	Summary of evidence		
Active use of online connections can reduce some negative mental and other health effects created by isolation policies (15)	Existing studies point towards small positive effects supporting this claim, but the number of studies is insufficient. One longitudinal study found small-to-moderate effects in the real world	Tested in real-world or field studies	Positive	Small	Articles reviewed: 15	Sample range: 110–6,523	
					Average review time: 16 h (spread over 3–10 days)	Mean sample: 2,062.8	Median sample: 1,344
Norms of prosocial behaviour are more effective when coupled with the expectation of social approval and modelled by in-group members who are central in social networks (4)	Evidence generally supports the notion with a medium-to-large positive effect. However, the available studies assess the general effect of norms, not the specific context stated in the claim	Empirical evidence (such as surveys, laboratory experiments and controlled settings)	Positive	Large	Articles reviewed: 45	Sample range: 52–108,075	
					Average review time: 10 h (spread over 2–5 days)	Mean sample: 10,709.3	Median sample: 2,006.5
A shared sense of identity or purpose can be encouraged by addressing the public in collective terms and by urging ‘us’ to act for the common good (1)	Claim is generally supported; however, it lacks real-world assessments with observable outcomes. Evidence mostly stems from survey data and online experiments	Empirical evidence (such as surveys, laboratory experiments and controlled settings)	Positive	Small	Articles reviewed: 54	Sample range: 130–910,006	
					Average review time: 20 h (spread over 2–15 days)	Mean sample: 25,532.3	Median sample: 729.5
Given the importance of slowing infections, it may be helpful to make people aware that they benefit from others’ access to preventative measures (8)	Clearly supported by limited evidence available, although best-evidence focus tended to be on intentions rather than true behaviours	Empirical evidence (such as surveys, laboratory experiments and controlled settings)	Positive	Small	Articles reviewed: 5	Sample range: 134–1,373	
					Average review time: 2 h (spread over 1 day)	Mean sample: 913.0	Median sample: 1,232

Overview of ratings and assessments for four claims (1, 4, 8 and 15). In addition, compilation and screening of articles were estimated to have taken over a total of 200 h. Note that summaries of sample sizes included any studies that included evidence and a rating, irrespective of overall influence on summary assessment. Articles may be double-counted if they were directly relevant for multiple claims.

**Table 3:** Evidence assessments for two claims on trust and leadership

Claim (number)	Evidence	Level	Direction	Effect size	Summary of evidence		
Identifying trusted sources (for example, local, religious or community leaders) that are credible to different audiences to share public health messages can be effective (2)	General support for the claim with a medium effect size from survey data in different samples and some applications in the real world. The core claim is generally supported by the evidence	Replicated real-world or field study evidence	Positive	Medium	Articles reviewed: 40	Sample range: 372–1,429,453	
					Average review time: 19 h (spread over 2–10 days)	Mean sample: 46,892.5	Median sample: 1,765
Leaders and the media might try to promote cooperative behaviour by emphasizing that cooperating is the right thing to do and that other people are already cooperating (3)	Evidence for the claim stems mostly from correlational data and few experimental studies reporting small but rather inconsistent effects across contexts and outcomes	Empirical evidence (such as surveys, laboratory experiments and controlled settings)	Positive	Small	Articles reviewed: 16	Sample range: 52–484,239	
					Average review time: 13 h (spread over 8–10 days)	Mean sample: 40,719.5	Median sample: 1,004

Overview of ratings and assessments for two claims (2 and 3). Articles may be double-counted if they were directly relevant for multiple claims.

**Table 4:** Evidence assessments for four claims on messaging and language

Claim (number)	Evidence	Level	Direction	Effect size	Summary of evidence		
Leaders and members of the media should highlight bipartisan support for COVID-19-related measures, when they exist, as such endorsements in other contexts have reduced polarization and led to less-biased reasoning (5)	The one reviewed paper directly testing the claim generally supports it, finding that bipartisan policy endorsements reduce polarization in views of, and increase overall support for, COVID mitigation policies	Empirical evidence (such as surveys, laboratory experiments and controlled settings)	Positive	Small	Articles reviewed: 12	Sample range: 350–10,699	
					Average review time: 7 h (spread over 1–10 days)	Mean sample: 2,724.2	Median sample: 1,995
There is a need for more targeted public health information within marginalized communities and for partnerships between public health authorities and trusted organizations that are internal to these communities (6)	Empirical evidence for the core of the claim exists; however, there is little evidence available that tests the effectiveness of the suggested approach. Existing studies have suggested a small positive effect of targeted messaging	Empirical evidence (such as surveys, laboratory experiments and controlled settings)	Positive	Small	Articles reviewed: 19	Sample range: 54–140,184	
					Average review time: 12 h (spread over 3–8 days)	Mean sample: 16,758.6	Median sample: 991
As negative emotions increase, people may rely on negative information about COVID-19 more than other information to make decisions. In the case of strong emotional reactions, people may also ignore important numerical information such as probabilities and the scope of a problem (11)	No empirical evidence that empirically tested the full claim. Existing evidence has focused more on the second part of the claim, not the first part, and might broadly point towards a small effect	Empirical evidence (such as surveys, laboratory experiments and controlled settings)	Positive	Small	Articles reviewed: 34	Sample range: 155–125,306	
					Average review time: 7 h (spread over 3–5 days)	Mean sample: 6,635.4	Median sample: 1,237
Use of the term ‘social distancing’ might imply that one needs to cut off meaningful interactions. A preferable term is ‘physical distancing’ because it allows for the fact that social connection is possible even when people are physically separated (10)	Besides a few small survey studies, support for the claim is purely based on theory and opinion. Therefore, no statement can be made about the potential effect size of this claim in application	No evidence has been identified, only discussion of the theory	NA	NA	Articles reviewed: 8	Sample range: NA	
					Average review time: 9 h (spread over 3–5 days)	Mean sample: NA	Median sample: NA

Overview of ratings and assessments for four claims (5, 6, 10 and 11). Articles may be double-counted if they were directly relevant for multiple claims. NA, not applicable due to neither of two studies with data being directly relevant to claim.

**Table 5:** Evidence assessments for claim 7 (five sub-claims) on messaging and language

Claim (number)	Evidence	Level	Direction	Effect size	Summary of evidence		
Messages that emphasize benefits to the recipient tend to be persuasive (7i)	Although some online experiments find limited support for the claim, the general picture is mixed. Existing applications in the real world indicate no general support for the claim	Replicated real-world or field-study evidence	Null	Null	Articles reviewed: 141 (claim 7 total)	Sample range: 208–163,627	
					Average review time: 22 h (spread over 2–14 days)	Mean sample: 19,599.1	Median sample: 3,964
Messages that appeal to social consensus or scientific norms tend to be persuasive (7iv)	The evidence is mixed, with online experiments and survey studies finding small positive effects of the suggested approach. However, these findings could not be replicated in an existing field study	Tested in real-world or field studies	Positive	Small	Articles reviewed: 141 (claim 7 total)	Sample range: 324–163,627	
					Average review time: 22 h (spread over 2–14 days)	Mean sample: 17,901.2	Median sample: 2,358.5
Messages that focus on protecting others tend to be persuasive (7ii)	Some real-world studies are available for the claim and point towards no effect. Evidence from online experiments has also been mixed, although some studies have found small positive effects	Tested in real-world or field studies	Null	Null	Articles reviewed: 141 (claim 7 total)	Sample range: 200–163,627	
					Average review time: 22 h (spread over 2–14 days)	Mean sample: 13,887.6	Median sample: 2,459
Messages that align with the moral values of the recipient tend to be persuasive (7iii)	No real-world studies with behavioural measures exist, but the existing evidence from survey data and online experiments has mostly suggested a small positive effect	Empirical evidence (such as surveys, laboratory experiments and controlled settings)	Positive	Small	Articles reviewed: 141 (claim 7 total)	Sample range: 246–24,682	
					Average review time: 22 h (spread over 2–14 days)	Mean sample: 5,019.8	Median sample: 1,683
Messages that highlight the prospect of social group approval tend to be persuasive (7v)	Few studies have tested this claim. The online experiments that do exist indicate mixed results	Empirical evidence (such as surveys, laboratory experiments and controlled settings)	Positive	Small	Articles reviewed: 141 (claim 7 total)	Sample range: 324–24,682	
					Average review time: 22 h (spread over 2–14 days)	Mean sample: 4,974.4	Median sample: 1,384

Overview of ratings and assessments for claim 7 (with five sub-claims), ordered by the level of evidence identified. Articles may be double-counted if they were directly relevant for multiple claims. Time estimates are given a single value for claim 7 due to the overlap of articles and the split in reviewers, but should be treated separately (that is, the total time spent reviewing all papers for claim 7 alone was over 100 h).

**Table 6:** Evidence assessments for four claims on culture, politics and misinformation

Claim (number)	Evidence	Level	Direction	Effect size	Summary of evidence		
Cultures accustomed to prioritizing freedom over security may also have more difficulty coordinating in the face of a pandemic (12)	Clear correlational evidence for an effect in different contexts and on different levels. Studies differ in the cultural dimensions assessed, including freedom–security, tightness–looseness and collectivism–individualism	Widely tested in real-world settings or field studies	Positive	Medium	Articles reviewed: 34	Sample range: 384–910,006	
					Average review time: 18 h (spread over 3–14 days)	Mean sample: 64,641.8	Median sample: 3,569.5
Preparing people for misinformation and ensuring they have accurate information and counterarguments against false information before they encounter conspiracy theories, fake news or other forms of misinformation can help to inoculate them against false information (9)	The application of this claim shows robust positive effects in online experiments and real-world applications, although effect sizes vary. Meta-analytic assessments of the effectiveness of the interventions exist	Replicated real-world or field study evidence	Positive	Medium	Articles reviewed: 60	Sample range: 102–33,480	
					Average review time: 15 h (spread over 3–6 days)	Mean sample: 4,340.9	Median sample: 1,554
Unmitigated political polarization will disrupt or create other negative effects on attempts to minimize or end the pandemic (14)	Robust findings for the effects of polarization in survey studies, but very few studies including manipulation or intervention. Context is very focused on the USA	Replicated real-world or field study evidence	Positive	Medium	Articles reviewed: 54	Sample range: 235–447,332	
					Average review time: 10 h (spread over 2–4 days)	Mean sample: 26,389.5	Median sample: 3,145.5
Fake news, conspiracy theories and misinformation will have a negative effect on vaccine hesitancy (13)^a^	Consistent evidence from survey data and correlational evidence for the claim indicating small-to-medium effect sizes	Empirical evidence (such as surveys, laboratory experiments and controlled settings)	Positive	Medium	Articles reviewed: 60	Sample range: 104–26,576	
					Average review time: 16 h (spread over 3–14 days)	Mean sample: 5,041.9	Median sample: 2,220

Overview of ratings and assessments for four claims (9, 12, 13 and 14). Note that the summaries of sample sizes included any studies that included evidence and a rating, irrespective of overall influence on summary assessment. Articles may be double-counted if they were directly relevant for multiple claims. ^a^The use of 'negative effect' here may create confusion for the intended meaning of claim 13; this is clarified in the Supplementary Information section “Notes for specific claims”.

Thirty-four studies report samples related to number of countries, studies, secondary datasets or other indirect observations. Sample sizes of the 463 original data studies included were large. The mean sample size of 418 papers specifically involving human participants was 16,848 (median of 1,699), with individual studies ranging from 52 to 1,429,453 participants. We present somewhat conservative estimates for both mean and median by including only those specifically involving human participants. We do not include in these estimates three studies (with samples from 3.7 million to 654 million) that used social media posts or accounts. Many studies also provided only vague indicators (for example, “more than ten thousand”) or aggregated groupings (for example, numbers of states, provinces or countries). Links in Data Availability give access to raw and interactive datasets for further exploration. However, only three studies reviewed had samples of fewer than 100 participants, whereas 279 had 1,000 or more. One-hundred and forty-two countries were included in one or more studies (see Evidence used for evaluations in Methods).

**Fig. 1:**
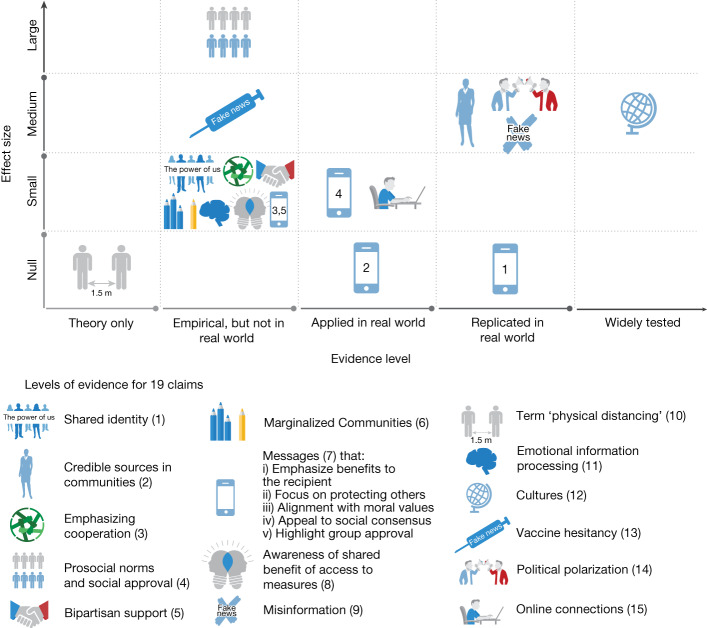
Reviewer-assessed effect size for each claim and the qualitative rating. The *y* axis shows the reviewer-assessed effect size for each claim; the *x* axis shows the qualitative rating (from theory only to widely tested). Each set of claims is represented by a different icon. Most claims were confirmed as having small-to-medium effect sizes, including those tested and replicated in real-world contexts. The strongest finding is indicated by the globe on the right, near the top, which shows the claim about culture (see Table 1) was widely tested in multiple studies and the results were consistent with the original Van Bavel et al. paper at roughly a medium effect size. A legend for each icon to represent the 19 claims is presented below the graph.

As depicted in Fig. 1, the direction of effect or correlation suggested by most claims were generally supported. Of the 18 claims that had at least some empirical evidence available for evaluation, 16 (89%) claims were generally supported in the direction of the original statement. Of the 16 claims that were supported by the research literature, ten were considered to show small effects, five were considered to show medium effects and one was considered to show a large effect. We did not find any meaningful effects in support of the remaining two claims, which stated that messages that emphasize benefits to the recipient (claim 7i) and focus on protecting others (claim 7ii) tend to be persuasive. This may be because several studies showed that there were moderators of which emphasis (self versus others) was more effective^44^.

Six claims (2, 4, 9, 12, 13 and 14) backed by empirical evidence demonstrated medium or large effects. Claim 4 (that norms of prosocial behaviour are more effective when coupled with the expectation of social approval and modelled by in-group members who are central in social networks) was mostly tested on observational data, but the effect sizes found in these data were notably strong.

Importantly, no effects were in the opposite direction from the original predictions. This means that no recommendations from the Van Bavel et al. paper led to a consistent backfire effect. Those 19 statements proposed behavioural domains that were likely to be of interest during the COVID-19 pandemic. Some claims were general about potentially relevant behaviours, whereas others were more prescriptive about potentially more effective intervention approaches.

Overall, our review indicates that the Van Bavel et al. article generally identified highly relevant topics of study in the pandemic and, to an extent, the direction of associated findings. In particular, it identified (1) relevant behaviours during the pandemic (both positive and negative), (2) likely barriers to mitigating the spread of the disease, and (3) major social challenges that would be faced by policymakers. The following text summarizes these in general groups, citing articles viewed by assessing teams as being most consequential for their final assessments. The ratings for each are specified in Tables 2–6.

All behaviours studied, whether specific to a claim or not, are listed in Table 7. We have included this table as a reference in the future for considering behaviours to expect or target.

**Table 7:** Behaviours directly studied in the initial literature review

Washing hands
Standing 6 ft or 2 m apart
Staying at home
Reducing visits to the store
Wearing a mask
Wearing a correct mask
Wearing a mask correctly
Wearing a best-fitting mask
Avoiding crowds
Using public transportation
Exercising at home
Exercising outdoors
Dieting during isolation
Consumption during isolation
Using the Internet
Using social media
Sharing public health information
Sharing misinformation
Getting tested
Testing at home
Visiting nursing homes or the elderly
Getting vaccinated for COVID-19
Refusing a COVID-19 vaccination
Delaying a COVID-19 vaccination
Getting a second shot (of two doses)
Getting a COVID-19 booster
Getting an influenza vaccine
Choosing single-shot doses
Using fake vaccine cards
Using unsupported treatment
Working remotely
Using mental health services
Going to hospitals
Postponing treatment
Spending stimulus money
Attending school remotely
Attending school in-person
Following or ignoring guidelines
Volunteering
Promoting cooperation
Promoting division
Messaging on social media
Using dating apps
Moving home with family
Isolating within home
Joining the military during the COVID-19 pandemic
Recycling and plastic consumption during the COVID-19 pandemic
Death^a^ and suicide
Downloading or using tracking apps
Trust^b^
Prosociality^b^
Intentions^b^
Beliefs^b^
Preferences^b^
Polarization^b^

^a^Death was a heavily studied outcome that was linked to both COVID-19 and the behaviours listed. There was debate as to whether it should alone count as a behaviour, so we included it for readers to decide. ^b^Behaviour-adjacent latent constructs that were commonly measured but are not possible to assess with simple binary, objective measures.

## Sense of identity

Four claims made in 2020 focused on how social identities would be highly relevant during the pandemic, particularly how they aligned with either community benefits or social norms. These expectations generally appeared to be accurate (see Table 2), with scores of studies concluding that connectedness with communities or aligning with morals were a predictor of behaviours and efforts to control the spread of illness^45–60^. However, one challenge that is typically present for research on subjective and latent constructs such as identity, prosociality and connectedness is that most research was conducted through surveys. Few studies attempted to isolate the causal effect of identity on pandemic behaviours, and no experimental studies manipulated identity or the sense of collective purpose in a real-world setting. In some cases, well-powered studies directly assessing the claims were limited to asking about intentions to receive vaccines^60^. Although such findings are very valuable, there is clearly additional benefit in validating those findings in consequential settings, or even carrying out retrospective studies to determine whether behaviours or infections were measurably associated with connectedness where studies were conducted.

## Trust and leadership

There was a large amount of evidence from peer-reviewed research on the role of leadership during the pandemic. Two claims from 2020 specifically outlined expectations for how trusted sources and leadership may be relevant to promoting public health guidelines. There was a substantial amount of research (see Table 3) supporting both of these expectations, although the best-quality evidence from consequential settings was replicated only in relation to the claim that the most effective messaging comes from trusted sources^61–66^. Consistent with original expectations, evidence has supported the value of highlighting the cooperation of other people to promote health behaviours, although evidence was limited to surveys and correlational studies.

## Messaging and language

Perhaps the most widely studied topic during the pandemic was public health messaging. This was clearly anticipated by Van Bavel et al. as 9 of the 19 claims explicitly discussed the role of messaging and language in developing effective public health interventions. Not surprisingly, this also produced the most heterogeneous set of evidence ratings (see Tables 4 and 5). Observational studies in natural settings concluded that messages directly emphasized benefits to individuals or protecting others had no measurable effect on behaviours^67^. There was some evidence for the effect of benefit-based approaches on behavioural intentions, although some studies suggested that self-benefit versus other-benefit messages were differentially effective for different types of people. Although benefit-based messaging has been found to be effective in general^68^, it is possible that there may be limits to its effect on behaviour in the context of novel health threats where the benefits of preventative behaviours are not well established and recommendations are evolving.

Messaging related to partisan concerns was also widely studied, although not often in consequential settings. For the studies with the most policy-relevant evidence, messages emphasizing consensus and general agreement about public health behaviours were more effective in promoting these behaviours than those considered to be polarizing or partisan in nature (in survey studies)^69^. A small number of related studies in the context of marginalized communities has found that direct engagement and direct messaging were more effective^70^.

Claim 10 on the use of physical distancing being preferred to social distancing yielded no evidence. Although eight articles were identified and required some time to review, none included direct evidence of any effect on making this change, and we have excluded it from Table 4.

## Social cohesion and misinformation

Claims specifically related to polarization and flawed sources of information were widely validated, with some caveats^71^. Across more than 200 published articles, polarizing and disingenuous messaging were consistently associated (see Table 6) with negative outcomes in terms of the effectiveness of public health interventions^72^. However, direct causal evidence was relatively scarce^73^. Studies with the highest levels of evidence have validated these patterns in consequential settings, often with medium effect sizes, indicating that greater division in messaging and lack of social cohesion were associated with lower effectiveness of public health messaging^48,73–85^. Encouragingly, inoculating against manipulation techniques^86^ and prompting users to consider accuracy before sharing news^87^ have some positive effects. Again, both lines of study would benefit from replications in consequential settings, with some additional validating work on this emerging after our review started^88^.

## Major themes not explicitly assessed

Several themes emerged during the search phase of this project that were discussed by Van Bavel et al. but not necessarily formalized in terms of a specific claim. These included the clear relevance of threat and risk perception, the role of inequality^89^ and racism^90^, skepticism towards science^91^, incentivizing behaviours beyond simply describing benefits (for example, by providing financial rewards for vaccination)^92–94^ and the absence of clear leadership^95–100^.

## Threat perception

Although ignoring threats and risk were concerns raised in the 2020 article, the statement that we assessed as a claim (11) did not yield a substantial amount of evidence to review. However, this was not because there was an absence of evidence relating to the general issue of threat perception. In fact, substantial research indicated that threat perception — and wilful decisions to ignore risks to self and others — were a major factor during the pandemic^101–103^. However, we chose not to create an additional generic claim to assess evidence related to this, not only to maintain consistency in the method but also because much of the research on this topic has been heavily associated with the polarization, messaging and misinformation themes. Still, there is clear and compelling evidence that deliberate decisions to ignore health information had negative impacts during the pandemic^102,103^.

## Nudging

Nudging was a widely attempted method for behavioural interventions during the pandemic. Huge increases in attempted nudges have arisen since 2019, largely due to the highly behavioural nature of pandemic policies. Although not explicitly framed as a claim in the 2020 article, nudging was highlighted as a practice likely to have a substantial bearing on pandemic-related behaviour.

Overall, interventions presented as nudges had mixed effectiveness during the pandemic. Encouraging evidence found that simplifying choice architecture and making options salient (for example, through personalized text messages), as well as making it easy to become vaccinated led to reductions in vaccine hesitancy^104,105^. The same has been found for improving availability of locations to receive a vaccine^101^. Accuracy prompts have also shown some promise as nudges that aimed to limit sharing of misinformation^87,106^, although replications have found generally small effect sizes for these nudges^87,107^. However, attempts at making use of lotteries to increase vaccination rates had no overall effect^92^, along with studies reviewed in claim 7 on messaging, which also had little impact^67^.

Because of the extreme number of trials, there is no single summary assessment that would appropriately cover the highs and lows, or complexity, of nudging during the pandemic. Several systematic reviews^108,109^ have explored the overall effectiveness of nudges, and more narrow systematic reviews have considered the effectiveness of nudges that target specific behaviours, such as vaccination^105^. In light of this mixed picture, we strongly encourage focused systematic reviews of all nudging carried out in the context of COVID-19 and urge nuance in determining which nudges work and which do not (treating them as equivalent does not seem to be supported by the data).

## Stress and coping

Unfortunately, the fear that isolation and lack of social connectedness would lead to a pandemic of mental illness largely played out^110–112^. Although much of daily life around the world adapted to major changes, the risk of prolonged and severe effects on mental health were widely identified, with large increases in depression, anxiety, stress and other common mental disorders reported globally^113^. In some cases, these effects were moderated (or at least attenuated) by being isolated along with close others^114^, whereas other studies have found dramatic increases in intimate partner violence and violence against women^115,116^. Some positive mental health outcomes had direct links to collective mindset and perspective^117^, although not able to circumvent all aspects, consistent with the 2020 article. Those patterns indicated a need to take more multidimensional approaches to well-being and mental health to find opportunities not only to treat or prevent illness but also to promote positive outcomes^118^.

## Major pandemic behavioural themes

Although matters such as polarization and vaccine hesitancy were discussed in the 2020 article and turned out to be clearly relevant, other themes not specified originally have been widely studied. For example, political divisions were not the only reasons individuals refused or delayed vaccination^119,120^ as there has also been evidence of general wilful refusal to follow public health guidelines (whether masking, social distancing, isolating when sick, avoiding unnecessary travel, vaccine hesitancy, and so on) in some individuals^121^. In this regard, explicit, manifest behaviours based on demographics and individual differences^122^ should also be reviewed as they have not been covered here. Those patterns are not inconsistent with perceptions, beliefs and social division, but we have not explicitly assessed that evidence. Even though some evidence pointed to the benefits of communicating good and effective policies directly to the public^123^, more needs to be done to explore how to achieve this when there is active, deliberate intent to criticize and disrupt those policies, without inadvertently giving greater visibility to those disruptive forces.

Other major themes not covered in Van Bavel et al. include more specific predictions about what outcomes may be associated with behaviours or policy interventions. For example, although there has been some mention of isolation impacting mental health, volumes of research looked at how school closures^124^ and curfews^125^ might influence children by limiting opportunities for interaction, playing and development, weighed against their likely effect on mitigating the spread of illness. Similarly, beyond social media, ways to address isolation might have involved better ways to engage communities in volunteering^126^ or other civic contributions for those that desired a more active role during periods of extended isolation.

Another theme not discussed was how traditional forms of mass media might have undermined the potentially helpful role of descriptive norms by giving disproportionate attention to anti-vaccination, conspiracy and other beliefs that did not reflect expert or even majority opinion in the general public^53,127^. Many countries, particularly those covered in the original article and where evidence was available for this paper, had vaccination rates above 70% (and sometimes above 90%). In these settings, messaging that focuses on the problem of vaccine refusal could mean giving a minority behaviour the same amount of attention as facts and evidence about widespread uptake and the benefits of vaccination^65^. In this regard, efforts of academics and public health officials may be thwarted if media policies around ‘equal coverage’ are implemented in ways that amplify false norms and harmful, fringe ideas, given how easily it is to manipulate or control narratives that are not rooted in evidence.

## General discussion

We approached this research with an appreciation that throughout the pandemic, especially early on, decisions had to be made on the basis of imperfect evidence (see ‘Building policy from imperfect evidence’ in the Supplementary information for further discussion). Our assessment focused on the quality, generalizability and policy relevance of available data rather than the average effect size of research related to the claims, using an expert-driven assessment of claims rather than formal statistical meta-analysis.

Our two teams of 72 total reviewers assessed 747 scientific articles covering 19 highly influential claims made in 2020 about human behaviour in the pandemic. Of the 747 articles, 463 studies included original empirical evidence and 418 had human participants (mean sample size of 16,848). Two independent teams evaluated the studies available for each claim (see ‘Author contributions’ for specific lists). Both teams found evidence in support of 16 of the 19 claims (84%), with no evidence available for one claim. For two claims, teams found only null effects. Overall, our review found that the Van Bavel et al. article generally anticipated meaningful topics that became relevant for research during the pandemic, and in the majority of cases, the direction of their associated research findings. In particular, it identified relevant behaviours during the pandemic (both positive and negative), likely barriers to mitigating the spread of the disease and major social challenges that would be faced by policymakers.

Aside from cultural effects on policy effectiveness, the most strongly supported intervention claim was how combatting misinformation and polarization would be vital to promote effective public health guidelines. Effective messaging, particularly in the form of engaging trusted leaders and emphasizing positive social norms, was also heavily supported in the literature. Broadly speaking, survey data strongly supported how critical it is for policy to understand collective behaviour, shared values and effects on marginalized populations to be effective at minimizing harms during a pandemic.

We strongly endorse a full systematic review of all behaviours studied during the pandemic, which would map not only what behaviours were observed (or not) and across how many studies but also where and through what methods. However, we also share the concern that many studies carried out during the pandemic, particularly in the early stages, had low power^42,43^. That has produced problematic meta-analyses, which was one reason why we chose an alternative approach.

## Applying lessons for science and policy

To make use of additional evidence that stemmed from this very broad reading of available literature, but which did not apply only to a single claim, we have consolidated critical recommendations for science and policy in Table 8. The purpose is to help researchers and policymakers respond to future pandemics, disasters or other exogenous shocks. These range from largely scientific practices, such as more study of global populations, and being more specific in formulating testable questions, to primarily policy topics related to communications and managing public uncertainty. Some of these recommendations may appear obvious or generic, but were not universally adopted in the studies that we reviewed.

**Table 8:** Recommendations for researchers and policymakers

Recommended future applications for science and policy		Summary	Example/application
	Formulate testable claims	Many prospective articles did not specify the directions of anticipated effects or identify causal factors. Others proposed complex components that were too nuanced to test. Meaningful precision in specifying claims and hypotheses would make it easier for practitioners to trial and incorporate evidence into policies	Prospective recommendations often indicated behaviours that might be ‘important’, but this creates an arbitrary standard as anything could conceivably be determined important or unimportant depending on the desired interpretation
	Study non-WEIRD populations	Recommendations made on the basis of evidence from WEIRD samples should not be uncritically generalized beyond these populations	Countries may need specific, local strategies that differ from those implemented in countries where much of the research has been done, because of differences in socioeconomic, political and macroeconomic conditions
	Think inside-out and outside-in	Claims from Van Bavel et al. primarily focused on the likely relevance of beliefs, perceptions, identity and other latent constructs. Research during pandemics should also focus on knowing what behaviours are most critical and the best ways to promote them, as well as identify interventions that consider structural contexts, not only psychological constructs	Early-stage recommendations from behavioural science in future pandemics should cover latent constructs (for example, identity, perceptions and norms), objective behaviours (for example, getting vaccinated and wearing a mask) and systemic factors (for example, access to the Internet, availability of healthcare and local legislation)
	Avoid the ‘streetlight effect’ by researching what matters, not just what is easy	Research during the pandemic often focused on what or who was easy to study rather than on what was most pressing for public health or who was most affected by the pandemic. Behavioural scientists should collaborate with practitioners to develop ways to make sure research resources are deployed where they will have the greatest impact. The most-studied populations in the pandemic were those with easy, stable access to the Internet. The most widely studied topics appeared to be those that could be tested through online surveys	Hundreds of studies on messaging were carried out online in WEIRD populations, but these did not demonstrate overall higher impact findings (in this review, at least)
	Test your assertions or programme evaluation	Many articles from 2020 made strong predictions that were not tested. The lack of clear validation or rejection of potentially influential policy interventions may allow some ineffective policy interventions to crowd out or divert resources from potentially more effective policy interventions	There was very little research studying whether the term ‘physical distancing’ would have more positive effects than ‘social distancing’
	Amplify according to evidence	The interventions getting the most attention were not necessarily those best supported by the most evidence. For example, correlations based on observational data were interpreted as if they were causal	Handwashing was widely promoted as a strategy for stopping the spread of COVID-19, yet study effects were small to null, particularly compared with masking, isolation, distancing and vaccines
	Precision, error, uncertainty and reality checks are always important	During the pandemic, public health agencies relied heavily on mathematical models with implausible assumptions about human behaviour. Behavioural scientists can improve these models by focusing on risk perception, innumeracy, noise, uncertainty and barriers to health behaviours (for example, access and costs)	When building models predicting behaviours such as vaccine uptake and isolation, factor in deviations from expectations based on practical, psychological factors
	Highlight null results	There is as much to learn from effective as ineffective interventions. Failures and backfire effects warrant more visibility to reduce attention to and spending on harmful or wasteful policies	A field experiment showing no significant effect of geo-targeted vaccine lotteries received very little coverage and influence on public policy
	Consider larger context	Research findings may vary depending on national, subnational and other local settings. Translating to policy interventions may require substantial adaptations to replicate effects	Appeal to national identity in liberal versus authoritarian regimes will probably differ in its behavioural consequences and ethical implications
	Do not overcommit too early	Although there are understandable pressures to issue guidelines quickly, establishing a policy position on poor or little evidence can lead to greater costs in the long term	Early guidelines in some countries suggested that wearing masks would not minimize COVID-19, but subsequent evidence has pointed to their effectiveness

Recommendations are ordered from primarily scientific to primarily policy, although to some extent, each recommendation applies to both.

In light of the challenges discussed, we offer two additional recommendations. These are specifically relevant to future public health emergencies but also apply broadly towards advancing the readiness of scientific evidence being applied to policy.

## Follow surveys with field research

Behavioural scientists, including those studying the pandemic, often use online data collection tools where there is substantial control over the treatment and, more generally, the research environment. We do not discount the privilege or benefits of having access to these tools or their contribution to foundational evidence that could be deployed rapidly. Online survey experiments could also offer insight on causality that, for example, descriptive or correlational field studies lack. Moreover, it is particularly encouraging that so much of that evidence converged, and this further clarified the added value of having access to those resources.

Despite those benefits, we recommend seeking opportunities to progress faster from concept testing to real-world testing and implementation in future crises. In the earliest days of the pandemic, there was no clear way to do this, and large numbers of studies were derailed, postponed, abandoned or forced to be modified substantially. This also presented challenges for validating evidence generated early in the pandemic (both at that time and during this review). In addition, new research designed to speak to the crisis had to be conducted using the tools available.

It is commendable that so much valuable and applicable social science emerged despite the chaotic circumstances, loss of resources and the uncertainty in the early days and months of the pandemic. Nevertheless, of the 518 studies given a ratings assessment as part of the present exercise, more than 400 were empirical studies conducted in laboratories or online settings (which may have also been skewed by both the nature of the claims and disciplines of individuals identifying relevant studies). Unfortunately, this also meant that many claims were only ever tested in surveys. Rather than this being a criticism, we state this as a strong encouragement to seek, promote and fund partnerships that can function in consequential settings, even (and perhaps especially) in the face of public emergency.

## Forge alliances

There are myriad challenges to linking scientific research to real-world practice, which were amplified during the pandemic. Although academics and institutions found ways to overcome these, practical constraints were evident throughout, whether based on resource and personnel limitations, or threats to health and safety. Other limitations may have included simply not knowing the appropriate communication channels to link researchers and policymakers.

In the future, we encourage academics that have not previously worked in such consequential environments to proactively engage with organizations delivering public services to find out where and what input they would value^128^. This will help to develop partnerships with local government offices^129^, hospitals^130^, banks^131^, schools^132^, local military units providing emergency personnel^133^ or other potential end-users of scientific evidence. Surveys are not a replacement for studying real behaviours in the field, such as blood donations, vaccines, assigning volunteers, facilitating remote work, keeping people safe while shopping or voting. Furthermore, researchers should recognize the potential for policy impacts at many levels: do not overly focus on highly visible policymakers or government employees who are difficult to access and may lack the subject-specific expertise to recognize all relevant research^134^.

For policymakers, managers, teachers and other institutional leaders, we also strongly recommend opening lines of communications with academics that research your professional area. Professionals seeking to apply insights generated can experience frustration at the reluctance of researchers to offer practical advice for evidence-based policies. Academics are typically not trained in ways to bridge the science–practice–policy gaps, and there are opportunities for impact in the future that may be in place if institutions also take the initiative to engage experts before emergencies. This is also a message for funding bodies: invest in initiatives that support cross-domain collaboration and translational research activity.

Both parties may feel reluctant and uncomfortable about such a collaborative effort, making them hesitant to consider working together as a viable option. Adopting a broader set of tools and research contexts would help both sides to see how they can collaborate more productively by expanding and applying their knowledge of important psychological phenomena and behavioural mechanisms in practice.

## Limitations

Tables 2–6 include multiple aspects of the evidence review, including the ratings, direction, effect sizes and a summary note. These were each included because no single rating or value can fully reflect the many dimensions of each behavioural domain. We do not intend or claim to offer a perfect ‘score’ of evidence or research during the pandemic. Instead, our goal was to provide a general assessment of evidence to guide future research and policy applications. Although some policymakers were asked to participate in this study, our focus was largely on synthesizing academic expertise to inform policy. We therefore note that, at the highest level, evaluations may prioritize scientific perspectives over insights most relevant to decision-makers and practitioners. Multiple, detailed discussion on the limitations of the approach, findings and interpretations of the work is provided in Supplementary Methods.

## Conclusion

Despite the absence of a consensus approach to selecting and synthesizing evidence, scientific research has a valuable role in public policy. The present study evaluated researcher claims and recommendations at the start of a global crisis. The synthesis of evidence suggests that researchers can be a viable source of policy advice in the context of a crisis and our recommendations highlight how this can still be improved across scientific disciplines.

Our synthesis and evaluation also speak to the value of revisiting the claims (predictive, indicative or otherwise) that scientists make about events that are of substantial relevance to policy once there is sufficient evidence to assess their validity^12,24,135,136^. This has the capacity not only to contribute to the sort of transparency that builds trust in science and public health but also to directly inform the development of relevant knowledge and tools for the next pandemic or other crisis.

Our final recommendation is vital and is directed to all scientists, especially behavioural and social, as well as to policy institutions: do not wait until the next crisis to form partnerships. It is always a good time to build relationships between organizations, clinics, schools, governments, media or any institution with which there may be mutual benefits towards building effective policies. This enables us to develop a robust and relevant evidence base and to ensure that we marshal our collective energies and resources so that we are able to use science to best effect in the service of serving, protecting, promoting and prioritizing the well-being of populations.

## Methods

Our approach assessed evidence related to the central statements or hypotheses (claims) in the original Van Bavel et al.^3^ article. For the purposes of evaluation, we treated these claims as testable hypotheses, then rated the level, direction and magnitude of findings relevant to each claim. Evaluation was conducted by seventy-two evaluators, including authors of the original study as well as an independent group of behavioural scientists and policymakers. Their assessments focused on whether evidence from the first 2 years of the pandemic supported, refuted or left unclear the validity of the claims (see the online database available at https://psyarxiv.com/58udn for details).

### Claims evaluated

We evaluated the ten claims highlighted in Table 8 of the original article, as well as five additional claims made in the main text. Those additional claims related to behaviours, themes or policies that ended up being especially relevant during the pandemic, such as vaccination choices and the influence of political polarization, but which did not clearly overlap with one of the ten primary claims. All other claim-like statements in the text were either already covered in the original ten or were not precise enough to assess. One of the original ten claims actually comprised five distinct claims, creating a total of 19 claims.

Some claims were more general and not well suited to be treated as hypotheses, but rather as recommendations. For example, claim 6 (see Table 1) first suggests that targeted messaging and then that partnerships with community organizations are valuable. The first part of that statement is more suited to treat as a hypothesis, whereas the second part states only that community partnerships may be worth looking into, but with no implicit impact. In this case, we disregarded the second part and focused on evidence that could inform the first part.

### Evidence used for evaluations

We identified articles and reports used for the assessment through extensive systematic and manual searches by all evaluators, with the primary criterion being that they were publicly available before 1 June 2022. Searches included using the pre-formatted systematic review code produced by PubMED-NCBI for research specifically on COVID-19 (available at https://pubmed.ncbi.nlm.nih.gov/help/#covid19-article-filters), as well as checking preprint servers (OSF, PsyArXiv and SSRN), multiple repository search engines (Google Scholar, PsycInfo and EconLit), crowdsourcing with forms to share articles (on social media and through targeted email lists) and snowballing of relevant articles (including articles that cited the original paper). There was no restriction for locations or language (see later for a discussion of the ways in which diversity of authorship enabled broader searching). This approach yielded approximately 3,000 articles initially. In a triage phase, team members checked for relevance of articles, removing duplicates and articles that did not meet the criteria (such as articles not relevant to the pandemic, being published outside the inclusion window or simply not relating to any of the 19 claims). Most removals were due to articles that were clearly not relevant but had been submitted for general relevance to behavioural science during the pandemic. Those articles were typically easy to identify; any ambiguous articles were left in to allow the full reviewers to determine relevance later. After that process, 747 articles were used in the initial assessment.

Across the 747 papers, 142 countries were represented (meaning at least one article existed where a country was studied directly). Total volumes by country ranged from 291 papers involving the USA to 1 (24 countries). Other countries with large numbers of papers included the UK (109), Germany (78), Italy (60), China (43) and Brazil (35). Full lists of the total geographical coverage of studies are included in the Supplementary information.

To ensure that we did not miss any major studies, all reviewers were asked to search for any potential additional articles after their assessments were submitted to the lead author (K.R.). Those articles had to meet the same publication deadline and were only included if they substantively influenced the overall assessment. One such article was identified^69^, whereas one set of interrelated studies was updated to include both original papers, letters to editors and responses to letters^45,67,104^. The latter three articles were added to the final list for posterity, but did not impact assessments. We therefore only refer to 747 articles unless stating otherwise.

Our aim was to identify the highest level of evidence available for (or null, or against) each claim, with higher ratings going to evidence from field research (that is, studies conducted outside the laboratory or survey), particularly highly powered evidence from multiple field studies and settings. This approach addressed two dimensions of evidence quality assessment akin to ecological validity. We use the word ‘support’ here when discussing evidence in which the original claim appears to be valid. However, major findings could also simply inform understanding, such as a highly powered but null finding. In this case, support indicates those findings that correlate positively with the intended meaning of the original claim.

We chose not to conduct a systematic review for several reasons. First, we were primarily interested in compiling evidence related to claims and reporting those. Conducting a systematic review would have required refining each of the claims to be more specific than originally written, which risks excluding a large amount of potentially relevant evidence^137^. There were also concerns that early-stage COVID-19 research was not suitable for meta-analyses due to being rushed, small samples (that is, underpowered), weak correlations or lacking appropriate methods, such as randomized controlled trials^42,43^. Therefore, we took a structured but more pragmatic and inclusive approach to selecting studies that addressed claims. This allowed us to cover a broader range of evidence for both insights and limitations of the work conducted during the pandemic.

Similarly, we asked reviewers in both teams to decide what evidence should or should not be included as part of the process. In a policy context, this means there may be disagreement over what evidence informs or does not inform a particular issue. Those disagreements cannot be resolved through selection criteria and extracting data alone (that is, disagreements would still occur in setting the selection criteria). We also wanted to minimize the possibility that a singular criterion confound (such as only permitting messaging on social media but excluding those in emails or letters) might overly bias expert assessments in the same direction. Because of this approach, we do not provide a PRISMA diagram, as each reviewer had different articles that they felt were or were not valid indications of evidence. Further details and limitations about this are provided in the supplement.

Finally, we included preprints in the study, allowing reviewers to determine the quality and robustness of material alongside material that was published after peer review. This decision was made because, for better or for worse^138^, preprints were extremely visible during the pandemic and often treated (at least by the public) as equivalent to published articles^139^. In addition, by including preprints, we reduced some concerns of publication bias by ensuring articles that may have not been published due to null findings could be considered.

### Evaluation teams

Two teams conducted the evidence review: 33 authors from the original paper^3^ were in one team and 39 independent reviewers made up the second team. The use of two independent teams was meant to minimize bias and increase the diversity of perspectives.

The lead author on this article was chosen based on experience in reviewing and reporting behavioural science in public policy contexts^140,141^, particularly in public health^142–145^, for having coordinated large-team research^143–145^ and for having led the development of an evidence standard^146^ for evaluating research for policy. To ensure full independence, the lead author — who was not involved in the original paper — only contributed to assessments by compiling all reviewer ratings, then reviewing articles identified as being ‘best evidence’. If any differences existed, the lead author then followed up to reconcile. In most instances, this involved discussion on what constituted ‘real world’, and the PI proposed a standard for discussion. There were no substantial disagreements with final ratings presented. This approach was intended to promote trust and integrity through transparency in assessment of previous work^35^ by involving multiple reviewers and many papers in the evaluation.

Reviewers represented institutions from more than 30 countries. There was some representation from every continent, although reviewers were predominantly based in Europe and North America. Searches spanned more languages than English-only articles, particularly white papers and other institutional reports on interventions during the pandemic that might inform the review. The claims that reviewers were assigned to assess were entirely confidential to reduce bias or influence, as were the names of all experts participating in the review. Because all reviewers were trained in the methods and coding system, names were visible in small groups. However, only the lead author knew who had been assigned to assess particular claims.

### Procedure

All reviewers followed a standardized approach to assessing each of the claims, which is included in the Supplementary information along with a brief tutorial. In each instance, reviewers received a set of articles assigned by the lead author. As the volume of papers varied substantially across claims (fewer than ten were found for claims 8 and 10; more than 100 were identified for claim 7), reviewers had different numbers of articles to review. For the larger volume claims, some procedural adjustments were made in which reviewers only assessed a subset of articles (a plan was established to address any issues created in the event someone then missed highly relevant material, but this only occurred once and was easily resolved). Each reviewer was required to read the articles in the list, noting four primary aspects:

1. Was the article relevant to the claim?
2. What was the level of evidence?
  No evidence, only opinions, perspectives, general theory or anecdotes
  Some empirical evidence but in limited settings (laboratories, surveys and online)
  A field study in a real-world setting
  Replicated evidence in field studies or other natural settings
  Wide-scale evidence from multiple field studies, policy evaluations or other natural settings
3. If there was any empirical evidence, was it in support (positive), null or against (negative) the claim?
4. If there was any empirical evidence, what was the general effect size (null–small–medium–large)?

After reviewing all articles, reviewers were asked to produce a summary of evidence for the claim, covering the same four themes. To focus the summary claim evaluation on the highest levels of evidence available, reviewers were asked to rate specifically based on the highest-quality studies reviewed. In other words, rather than averaging all available evidence (as in a meta-analysis), reviewers focused their summary assessments of the highest levels of evidence. For this work, ‘highest’ was defined by the 1–5 scale, supported in terms of statistical power, the appropriateness of method and scope (broadness of settings). This procedure is more relevant in a policy context, in which it is preferable to evaluate the highest-quality evidence available than to estimate average effects from all available evidence (all individual article ratings are available). Causality was not directly factored into the rating given that it is not typically weighted more heavily in policy, and because it might have sidelined rare-but-valuable field studies during the pandemic and only taken stock of well-controlled online experiments.

All reviewers were actively encouraged to assign their own ratings for articles and claims, and there was no attempt to force unanimity across raters. Despite this, average inter-rater agreement (percent of times within each article that reviewer scores agreed) was high (77.5%). This value is generally good, but especially so considering that some claims had so many relevant papers that it was not possible for the same reviewers to assess them all. In addition, several papers were rated by some assigned reviewers but not others when the reviewers disagreed regarding the relevance of the paper to the claim (those judging a paper to be irrelevant were not asked to evaluate it).

We emphasized effect sizes and direction of associations rather than statistical significance to address major concerns regarding validity and replicability. Relying only on *P* values would have increased the likelihood that publication bias and misrepresentation could have overstated evidence in the papers that we reviewed. Following well-known concerns^147,148^, our guidelines did not involve *P* values; only effect sizes were discussed given that they are more predictive of replicability^149^. This approach was largely supported by the consistently large sample sizes of studies included (see Tables 2–6).

Furthermore, including preprints in the assessment reduced the potential role of publication bias. Similarly, we included the direction of effects (positive or negative) to both clarify whether the finding generally agreed with the claim and to account for possible backfire effects, and the harmful side effects that might entail (for example, if a study showed a messaging intervention did have the expected effect, but also created more severe harms, it was possible for the assessment to reflect this).

Evaluations were submitted to the lead author; no reviewer was allowed to see other reviews. The lead author anonymized evaluations to a central coordinating team that checked evaluations for mistakes or inconsistencies (for example, ratings that did not align with the article noted as highest evidence or with the summary statement). All material from these processes have been compiled and posted in an interactive format for public use (https://tabsoft.co/3xZwIbD). Note that all values related to sample sizes have been provided as a general indication within and across claims, reflecting a simplified summary of studies reviewed (where samples were clearly reported). As discussed in ‘Evidence for 19 pandemic behaviour claims’, there can be debate as to what should be considered a sample size as well as challenges in extracting values. Since these were not the focus of the research, we provide them for general context and have posted the compiled spreadsheet for those that may wish to revisit or recalculate.

A detailed discussion of all procedures and their limitations is provided in the Supplementary information.

### Reporting summary

Further information on research design is available in the Nature Portfolio Reporting Summary linked to this article.

## Online content

Any methods, additional references, Nature Portfolio reporting summaries, source data, extended data, supplementary information, acknowledgements, peer review information; details of author contributions and competing interests; and statements of data and code availability are available at 10.1038/s41586-023-06840-9.

### Supplementary information

Supplementary Information This file includes Supplementary Methods and general notes on the process of potential relevance to future work, additional references, Supplementary Tables 1 and 2, tutorial for using evidence evaluations, manual for reviewer teams, the original claim wording and FAQs used for evidence review.

Reporting Summary

## Figures and Tables

**Extended Data Table 1:**
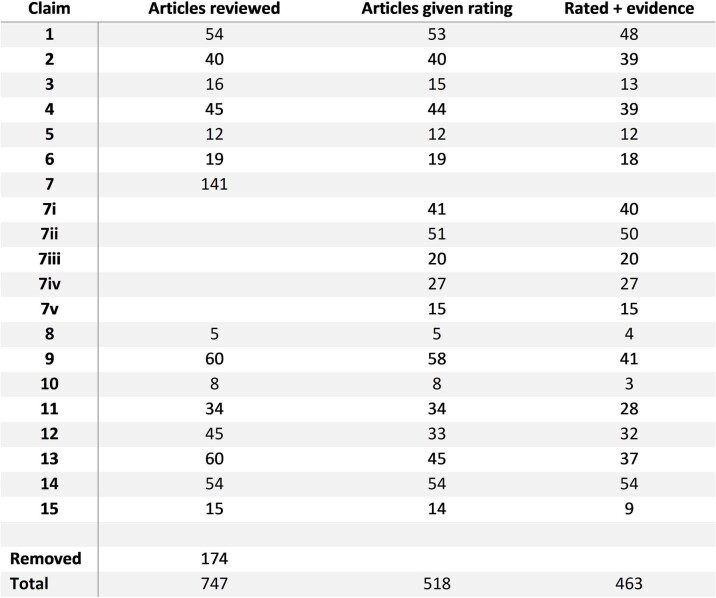
Articles reviewed with evidence by claim

Each row represents the articles reviewed by claim. Articles will not add up to the totals as 25 papers were initially reviewed under more than one claim. Of the 747 included after the triage phase (from over 3,000), 174 were later determined not to fit specifically to a claim. Of the 518 that received at least at least one rating, 118 were reviewed under more than one claim (the increase and largest impact was created by separating claims 7i-7v). Of those, 463 articles were deemed to have empirical evidence, which refers to the sample sizes described in the Main Text. Of those articles, 100 were reviewed under more than one claim. Also note that receiving a rating (with or without evidence) did not necessarily mean an article was deemed to validate or refute a claim. For example, though there were eight articles reviewed under claim 10, none of these were deemed to directly address the claim itself.

## Data Availability

All data and study material are provided either in the Supplementary information or through the two online repositories (OSF and Tableau Public, both accessible via https://psyarxiv.com/58udn). No code was used for analyses in this work.
