# Supplementary material for: A synthesis of evidence for policy from behavioural science during COVID-19
Source: Nature. 2023 Dec 13;625(7993):134–47. doi: 10.1038/s41586-023-06840-9 (PMC10764287; doi:10.1038/s41586-023-06840-9)
Supplement: Supplementary file 1 — This file includes Supplementary Methods and general notes on the process of potential relevance to future work, additional references, Supplementary Tables 1 and 2, tutorial for using evidence evaluations, manual for reviewer teams, the original claim wording and FAQs used for evidence review. [file 41586_2023_6840_MOESM1_ESM.docx]

**Supplementary methods**

The following sections include the methods of this project. Most aspects simply complement details in the main manuscript, with some notes on how plans were adjusted during the review compared to original plans. Most changes were administrative and there were no substantive changes to report otherwise. The detailed manual at the end is the precise material and procedures all reviewers were required to follow. Data from all articles reviewed are available via the sources listed in the main text (https://tabsoft.co/3CjCsOV).

Note that the format of each section looks very different. This is not laziness of the authors but a way to indicate how the material appeared in practice as closely as possible.

**General notes on the process of potential relevance to future work**

The following text presents notes that were compiled in advance of running the review, with only slight adjustments for clarification on timing.

*Evaluation scoring*

The evaluation scale used in this review ranges from 1 to 5 and follows a very simple progression of purely speculative work with no empirical evidence (1, Theory) to substantial evidence in real-world, consequential settings in multiple contexts (5, Impact). This scale was used explicitly to not appear as a perfect, all-encompassing metric for evidence, but instead offering a policy-informed approach to assessing and comparing against expectations made regarding proposed interventions. We also chose the this frame specifically because of how it would not give a misleading indication of objectivity or absolute standard, but instead, provide a qualitative level of assessment that must be accompanied by other factors.

Such issues in identifying best-quality evidence apply directly to policy decisions, which are ultimately subjective decisions. This is a direct result of the inability to offer a single, linear, and universally robust standard as well as the impact of external pressures and resource limits. Because of this, we are investing heavily in limiting that subjectivity by implementing a standard to at least produce consistency in *how* evidence is rated. In a perfect world, this might produce consistent assessments. However, in a more realistic world, it minimizes bias from the *process*, include a metric for users to interpret, and indicate the limitations of assuming there is any perfect way to determine policy. Doing so helped avoid overstating any claims of appropriateness for policy. Furthermore, by having multiple reviewers - and in this instance, multiple teams - for all papers and claims, we removed the capacity of a single individual to excessively influence a rating.

Additionally, NASA Technology Readiness Levels (TRL), and similar tools built or proposed on its premise, largely take a linear approach to development. However, scientific evidence is typically reliant on breakthroughs, which are hopefully followed by replications and ex post evaluations. Something can go from an untested theory to widespread implementation in a moment’s notice due to urgent policy demands. In reverse, major studies can produce negative effects, which would still be published. Product tracking in TRL assumes improvement or failure to progress; it is critical to highlight negative evidence whereas a negative product is typically dropped or at least isolated for future consideration. Finally, TRL-adjacent approaches tend not to be informed by actual policymakers, and appear to be written by individuals that are not familiar with making policy first-hand.

*Who is doing the evaluations?*

To ensure both a very high level of expertise in the ability to identify, compile, assess, and critique evidence while also addressing potential bias by incorporating impartiality, we included two evaluation teams independent of each other. All materials reviewed for the assessment to allow external evaluations (potentially using other protocols or even from adversarial teams) are included with this supplement.

The first evaluation team involved the original authors, who were primarily responsible for compilation of evidence related to the specific claims they proposed. To the extent possible, this team has no role in the design of the evaluation plan nor the guidelines being used to select papers, assess evidence, or summarize findings. To promote transparency, the methodology was developed independently from these authors (with the exception of general pragmatic aspects, such as efficiency in compiling papers), with only the exception of the two original leads, who shared feedback on drafted plans but were not involved in final decision-making. Similar approaches have been used for involving original authors in large-scale replications.

To build on this plan, the second evaluation team involved roughly 30 early career researchers and senior professionals from over 15 countries that were completely independent from the original article. Those reviewers have no affiliation to the authors of the 2020 article and were asked to seek additional papers for evaluation (to ensure potentially contradictory findings are not minimized from bias, among other reasons).

We originally intended to present the results from the two teams separately, but given most disagreements in the reviews were minor, this was dropped from the final publication. This was considered acceptable as other controls in the procedure were in place by having these two teams, such as ensuring reviewers would read the claim in two ways: as written and as intended. The independent team had only the text to work from and therefore would assess as a reader; the original authors were part of the writing and may have had a different perspective of seeing the intended meaning beyond the verbatim text. In the end, this did not appear to be a major issue, so we removed further discussion from the main text and only include this brief note about the general thinking.

For a general understanding of rater agreement beyond what is presented in the main text, in the initial evaluations, 56% of ratings matched perfectly with the consensus ratings reported in Table 2, and 84% were within one level of the consensus. In other words, working entirely independently, even before any direct communication to clarify differences, two independent teams were within 1 level of the final consensus rating 84% of the time. Furthermore, the average claim rating between the two teams was not significantly different, which we checked to ensure no concerns that original authors might have rated more highly than the independent team.

*Reflections on rating evidence*

To provide these insights in the most accessible way, the evaluation structure described the breadth, scale, impacts, and summary findings across a nascent but unprecedented volume of evidence. This structure is not perfect; to support those that may wish to further develop such a process, we offer general points on the development and assessment of evidence during the COVID-19 pandemic, as well as the relevant limitations of our approach along with ways to overcome these in future.

The two most critical recommendations involve a slight shift in the rating levels and having strict but useful definitions of what constitutes “real-world”. First, it may be better to adjust the evidence classification by treating ‘theory only’ papers as 0 out of 5, and then splitting empirical research conducted in controlled or survey settings into two categories reflecting different levels of evidence quality. This recommendation emerges from recent advances in scientific standards in behavioral sciences, which have shown discernible differences in the quality of studies conducted in controlled or survey settings (in particular: reliability, robustness, insight on causality, power, representativeness/sample quality, and replicability). That variability implies some studies are more appropriate for consideration in public policy than others and can be rated as such. For example, ‘low empirical’ evidence could be rated as 1, with more robust, higher-powered, evidence with higher insight on causality (that is not conducted in field settings) treated as 2. We found many examples of studies of each type (though would not have affected our summary claim ratings here).

The second recommendation stems from skepticism over the viability of a universal rule for establishing a threshold of controlled versus real-world or field study evidence. As a result, evaluations for policy may need to introduce a rubric for evaluating research *a priori*, but allow some flexibility for contentious examples. There may also be value in conducting reliability assessments of any classification that involves researchers, practitioners, and policymakers. We also note that our approach did involve multiple ways for reviewers to flag harmful side effects or unintended consequences of various interventions, whereas future applications may want to have more explicit statements in their guidelines for how to identify and assess these effects.

Further, the current rating system does not value evidence of causality more highly than correlational evidence. Thus, for example, evidence in support of a claim could achieve a Level 5 rating even without evidence that the hypothesized pattern is causal. Where applicable, future work on the rating system could integrate the strength of evidence for causality, perhaps as an additional dimension. Finally, as with most large-scale evaluations, all aspects of the quality of each individual paper are not examined (e.g., for the use of questionable research practices). However, the inclusion of a large number of papers for each claim including those in the so-called “gray literature” (i.e., papers that are not formally published) offsets these and other concerns (e.g., publication bias) to a degree. Future evidence assessments could also include a more detailed guideline on how to assess quality to minimize related concerns or this approach could be complemented by other strategies designed to assess publication bias (e.g., p-curves).

*Reflections on limitations*

Our overall approach is, we believe, especially helpful in domains where the volume of research is especially large, methods are particularly diverse, the concept is broadly defined, and quality of evidence is variable. The present application is intended to illustrate its utility for future assessment work. However, we do not intend the method to displace other approaches, such as meta-analyses and other forms of systematic review. Instead, meta-analytic and systematic review tools can complement each other. Because this may be practically unlikely in most cases, and because there are concerns about the use of meta-analyses for COVID research^1^, we encourage future work to carefully clarify when different review and meta-analytic techniques are most helpful for different evidence assessment goals.

*Administrative note*

All reviewers were asked to list claims they felt comfortable assessing. We required at least four reviewers per claim (two from each team) to ensure that allowing reviewers to self-select claims where they may have vested interests would not lead to potentially biased assessments. From those responses. The PI assigned all reviewers to a claim. Because some claims yielded only a very small number of articles, some reviewers were asked to take on two claims. A small number of noble reviewers also volunteered to go above and beyond, participating in multiple claims with large volumes of papers.

For transparency, we note that this approach was slightly different than the original plan, which was essentially a result of having a sufficient number of reviewers that we did not need to use such a complex review system. That approach, which was not used but did influence some of the final plan, was:

*All reviewers will be given access to 1-2 folders related to specific claims that we will evaluate. Reviewers from the original authors will be asked to focus on the claims that related to their expertise, typically only one claim. Independent reviewers will be asked to select which claims they feel competent enough to review but not biased on how they view the claim. All reviewers will be asked to announce conflicts of interest (independent reviewers cannot have conflicts with their claim; original authors are likely to, so we will be transparent about them but not disqualify except where necessary for the integrity of the study).*

In the end, all papers were reviewed by at least four individuals (two from each team), as were all claims. Claims with a greater number of papers had more reviewers, and all claim assessments were checked again by a central team and the PI. This approach was slightly different than originally intended (six reviewers for each claim), but this was only because it was not necessary for claims with only a small number of reviewers and generally uniform responses.

Reviewers were not be able to see scores or summaries of other reviewers during the process. Only after all reviews were completed, compiled, anonymized, and synthesized did reviewers see all responses. Those reviews are now publicly available at: https://tabsoft.co/3CjCsOV.

The first author was only a tie-breaker for final assessments reported in the manuscript, but had no role in any specific claim or article evaluation. Following the consensus table, all reviewers were allowed to comment on the assessments reported to ensure they were satisfied with the unified summary.

**Simplified, adapted PRISMA diagram of articles reviewed for each claim and overall ratings**

**
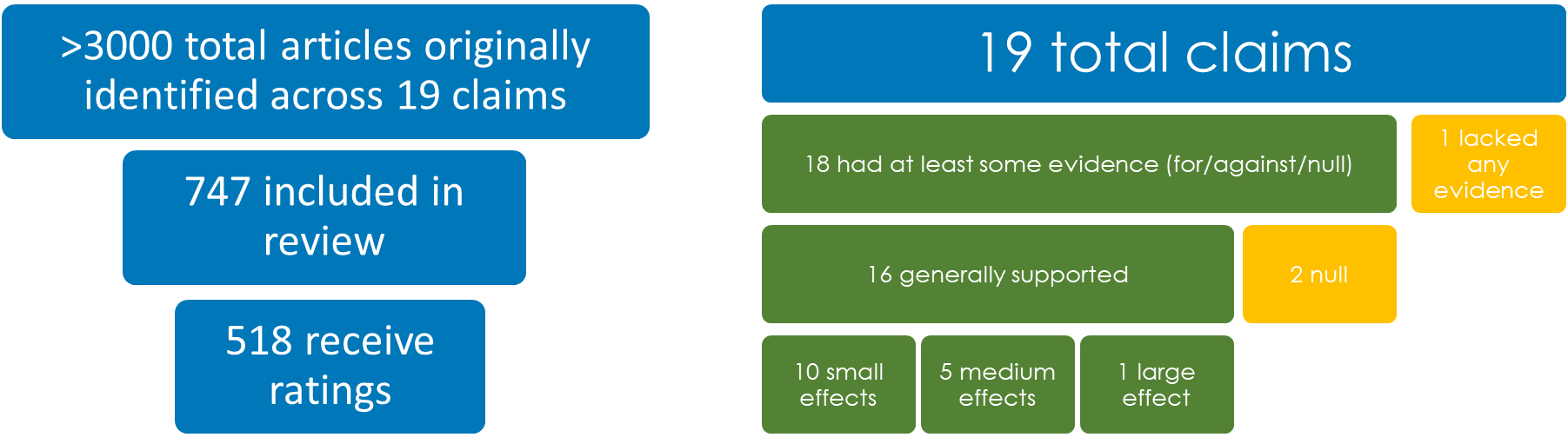
**

*Building policy from imperfect evidence*

Under ideal circumstances, major policy challenges would be easily defined and well understood, and the research needed to inform decisions would be available, robust, applicable, and perfectly informative. Even under ideal conditions, which the pandemic was clearly not, decision-makers still need to balance costs and benefits, as well as recognize that there may be varying risk and uncertainty associated with each possible outcome.

The current evidence synthesis was a major undertaking by over 70 researchers working in more than 30 countries, evaluating evidence produced during an emergency characterized by high uncertainty, volatility, and political division. The complexity of tasks associated with reviewing evidence along with the sprawling nature of the pandemic’s impacts on behavior (and vice versa) meant that several challenging decisions had to be made at each phase of the research process. For posterity, we highlight three key considerations created by those challenges, some of which are likely universally applicable when it comes to evaluating evidence in relation to policy. They speak to the general question of how academic research can best inform policy, and to the observation that this can have fundamental limits even in the best of circumstances.

*Error is hard to quantify*

One critique of the original article was that the evidence applied by authors was not necessarily field-tested in a way that would justify policy implementations during an emergency. The argument essentially noted the risks of a Type 1 policy translation error in which social and behavioral scientists recommend an intervention that does not work (false positive). However, the reverse of this critique is also a potentially harmful risk: should there be no attempts to use evidence-based messaging techniques during a public health emergency? It also reflects a lack of understanding of policy, resulting in a Type 2 policy translation error in which interventions might have been successful but were not used (false negative).

In practice, it may be harder to detect false negative errors, which leads critics to focus disproportionately on false positive errors. This can be problematic or even dangerous. For instance, smoke alarms are designed to be highly sensitive because the costs of Type 1 errors (false positives/false alarms) is much lower than the cost of Type 2 errors (false negative: an undetected fire that could lead to loss of life). As such, any policy decision should carefully weigh the tradeoffs between Type 1 and Type 2 errors, rather than focusing exclusively on the most salient risk.

Most policies cannot be perfectly informed by existing evidence because many public challenges are not well-understood until after they emerge and require action. Only later can these challenges and policies be properly evaluated. As we noted in the Methods (see *Evidence used for evaluations*), validating metrics for a scientific instrument involves comparing against external factors to ensure levels of external validity. However, in policy contexts, external validity may take years to assess with confidence (or, at least, to assess beyond anecdotal evidence). Yet most policy challenges demand immediate action that cannot wait for such evidence to emerge, as was clearly the case with COVID^2^. Future evaluations of evidence could include comparing results not yet available against what evidence was available for inclusion in this review, whether individual studies, meta-analyses, or Delphi expert panels^3^.

We also note that our assessment of evidence is a combination of a substantial number of indirectly related studies, which may each include forms of statistical and researcher error. Our approach minimized the multiplication of error by encouraging all reviewers to factor in the quality of studies to prevent conflating good evidence with unreliable findings and, in turn, limit the likelihood of false positives. This resulted in widespread agreement about the direction of findings for most claims. Moreover, this direction was predominantly positive (i.e., supportive of those claims).

There was less agreement about the size of effects. This increases the likelihood of Type M^4^ error in the consolidated assessment: the true magnitude of the patterns and effects may be different from those we report. Thus, Table 2-5 values should be considered indicative rather than absolute. The original plan for this evaluation was to split values based on whether reviewers were original authors or in the independent team, but there was no clear pattern of difference between these two groups of raters, suggesting a broad consensus. Accordingly, we presented a single value.

The more consequential distinction in assessments pertained to the classification of “real-world evidence.” This was a persistent challenge in assessing evidence because no clear rule is universally applicable for what counts as “consequential” or “real-world.” In some cases, reviewers were suggested (but not required) to use this example as a general guideline: ‘a survey that asks individuals if they intend to get vaccinated is not real-world; clinical data on the number of individuals getting a vaccine is.’ However, this instruction has two major limitations. The first is that there is a gray area between those classifications: if an individual reports getting vaccinated, should this be treated as real-world? The second is that more subjective and latent constructs such as mental health are almost canonically assessed via surveys. For example, an online survey of mental health and social media use may not be as objective as clinical admission for depression and a monitor on a phone that tracks social media use. However, the core material may not vary substantially in those instances. In this way, Type M error is also a concern, but it is one that policymakers and practitioners can appraise themselves in relation to their goals in a given context even if reasonable minds may disagree.

Future attempts at evaluation of policy-relevant evidence could incorporate reviewer calibration training to improve reliability, and could report whether that corrects some of these issues. However, we strongly discourage instructions that push reviewers to give a rating they do not feel is defensible in exchange for consistency. The ability to dissent is critical in policy discussions.

*Qualifications for evidence*

There is no perfect metric to assess evidence for application. Even with considerable structure and standards aiming for objectivity, the process is fundamentally subjective. For example, consider a 2% increase in the number of people getting vaccinated following an intervention built on community-specific trust. Irrespective of statistical significance, some may see this as too small an effect to be meaningful for future attempts, especially if it came at a large cost to taxpayers. Others, particularly those who believe that any potential for a saved life is sufficient, may see it as wildly successful.

Furthermore, it is not only the content of the material that will be important, but the source. A high-impact study may be questionable if it is published in, for example, a predatory or pay-to-play journal. However, even reputable journals can fall victim to junk science, as seen when two of the most highly regarded medical journals were forced to retract papers on hydroxychloroquine^5^. This is also further validation for our approach: the highest levels of evidence require multiple studies rated by multiple reviewers, which at least reduces undue influence of a single source.

Similar challenges also arose when it came to defining “behavior” versus more general concepts that were widely studied in research. For example, while getting vaccinated could be measured in binary choice terms, vaccine intentions and trust in science are more challenging to quantify and validate, though proxies of latent constructs are possible. We recommend parallel research that produces a more systematic and empirically informed threshold for establishing these classifications. Such work would be especially useful if it tested different guidelines for classification and assessed the reliability across a diverse set of expert reviewers.

*Limited evidence is not bad science*

One concern raised by reviewers during the evaluation phase was the substantial number of papers considered as empirical-only evidence (i.e., not tested in consequential settings). This may have been due, in part, to the very significant health risks of interpersonal interaction during a pandemic, putting researchers and participants at direct health risk. Moreover, it should be seen as validation that behavioral scientists were heavily committed to building an evidence base at conceptual, controlled, or non-consequential levels safely prior to rolling out potentially impactful and costly interventions across whole populations. As discussed, there is certainly a need to pivot away from online studies to working in settings with partners of all types, but a strong base of empirical evidence in more controlled settings is beneficial.

The existence of many theoretical or simple empirical studies is not a negative indication, but no accumulation of such papers could ever constitute fully validated evidence. Even with many converging findings from well-powered studies in controlled settings, claims were not assessed as “applied”. Contrarily, for claims where a large number of real-world studies were attempted, having many field studies and replications exist can equate to an overall claim being considered as having its impact widely validated. This differentiation is important: many standalone, consequential studies from different populations, countries, settings, and domains can indicate replicability and clarity on the globalizability of a behavioral construct^6^.

**References used in supplement only**

1. ‘It’s misinformation at worst.’ Weak health studies can do more harm than good, scientists say. https://www.science.org/content/article/it-s-misinformation-worst-weak-health-studies-can-do-more-harm-good-scientists-say.

2. Ruppel Shell, E. Act now, wait for perfect evidence later, says ‘high priestess’ of U.K. COVID-19 masking campaign. *Science* (2020) doi:doi: 10.1126/science.abf2811.

3. Lazarus, J. V. *et al.* A multinational Delphi consensus to end the COVID-19 public health threat. *Nature* **611**, 332–345 (2022).

4. Beyond Power Calculations: Assessing Type S (Sign) and Type M (Magnitude) Errors - Andrew Gelman, John Carlin, 2014. https://journals.sagepub.com/doi/10.1177/1745691614551642?url_ver=Z39.88-2003&rfr_id=ori:rid:crossref.org&rfr_dat=cr_pub%20%200pubmed.

5. Two elite medical journals retract coronavirus papers over data integrity questions. https://www.science.org/content/article/two-elite-medical-journals-retract-coronavirus-papers-over-data-integrity-questions.

6. Ruggeri, K. *et al.* The globalizability of temporal discounting. *Nat Hum Behav* 1–12 (2022) doi:10.1038/s41562-022-01392-w.

**Table S1. Overview of all claim ratings (1-5) by reviewer along with consensus/final rating**

| **Text** | **Low** | **High** | **Mode** | **Consensus** |
| --- | --- | --- | --- | --- |
| 12. Cultures accustomed to prioritizing freedom over security may also have more difficulty coordinating in the face of a pandemic.* | 3 | 5 | 3-4-5 | 5 |
| 2. Identifying trusted sources (for example, local, religious, or community leaders) that are credible to different audiences to share public health messages can be effective. | 2 | 4 | 4 | 4 |
| 7. Messages that (i) emphasize benefits to the recipient tend to be persuasive. | 2 | 5 | 3 | 4 |
| 9. Preparing people for misinformation and ensuring they have accurate information and counterarguments against false information before they encounter conspiracy theories, fake news, or other forms of misinformation, can help inoculate them against false information. | 2 | 4 | 2 | 4 |
| 14. Unmitigated political polarization will disrupt or create other negative effects on attempts to minimize or end the pandemic. | 2 | 4 | 2 | 4 |
| 7. Messages that (ii) focus on protecting others tend to be persuasive. | 2 | 5 | 3 | 3 |
| 7. Messages that (iv) appeal to social consensus or scientific norms tend to be persuasive. | 3 | 3 | 3 | 3 |
| 15. Active use of online connections can reduce some negative mental and other health effects created by isolation policies. | 2 | 3 | 2 | 3 |
| 1. A shared sense of identity or purpose can be encouraged by addressing the public in collective terms and by urging ‘us’ to act for the common good. | 2 | 4 | 2 | 2 |
| 3. Leaders and the media might try to promote cooperative behavior by emphasizing that cooperating is the right thing to do and that other people are already cooperating. | 2 | 3 | 2 | 2 |
| 4. Norms of prosocial behavior are more effective when coupled with the expectation of social approval and modeled by in-group members who are central in social networks. | 2 | 5 | 2 | 2 |
| 5. Leaders and members of the media should highlight bipartisan support for COVID-related measures, when they exist, as such endorsements in other contexts have reduced polarization and led to less-biased reasoning. | 1 | 3 | 1 | 2 |
| 6. There is a need for more targeted public health information within marginalized communities and for partnerships between public health authorities and trusted organizations that are internal to these communities. | 2 | 3 | 2 | 2 |
| 7. Messages that (iii) align with the recipient’s moral values tend to be persuasive. | 2 | 2 | 2 | 2 |
| 7. Messages that (v) highlight the prospect of social group approval tend to be persuasive. | 2 | 2 | 2 | 2 |
| 8. Given the importance of slowing infections, it may be helpful to make people aware that they benefit from others’ access to preventative measures. | 2 | 3 | 2 | 2 |
| 11. As negative emotions increase, people may rely on negative information about COVID-19 more than other information to make decisions. In the case of strong emotional reactions, people may also ignore important numeric information such as probabilities and a problem’s scope. | Empty | 2 | 2 | 2 |
| 13. Fake news, conspiracy theories, and misinformation will have a negative impact on vaccine hesitancy. | 2 | 2 | 2 | 2 |
| 10. Use of the term ‘social distancing’ might imply that one needs to cut off meaningful interactions. A preferable term is ‘physical distancing’, because it allows for the fact that social connection is possible even when people are physically separated. | 1 | 2 | 1 | 1 |

*One reviewer for claim 12 did not read all articles and gave a rating of 2, which was appropriate for the articles reviewed but did not include several papers that were of larger scale, power, and quality.

**Table S2. Total number of papers included in review by location**

| **Ordered by volume** | | **Alphabetical order** | |
| --- | --- | --- | --- |
| **COUNTRY** | **Number of studies** | **COUNTRY** | **Number of studies** |
| United States | 291 | Afghanistan | 3 |
| United Kingdom | 109 | Albania | 2 |
| Germany | 78 | Algeria | 6 |
| Italy | 60 | Andorra | 1 |
| Spain | 45 | Angola | 2 |
| China | 43 | Argentina | 18 |
| Canada | 42 | Armenia | 2 |
| France | 39 | Austria | 11 |
| Brazil | 35 | Azerbaijan | 3 |
| Poland | 35 | Bahrain | 1 |
| Mexico | 30 | Bangladesh | 13 |
| India | 29 | Barbados | 1 |
| Japan | 29 | Belarus | 3 |
| Netherlands | 29 | Belgium | 9 |
| South Korea | 29 | Benin | 1 |
| Turkey | 29 | Bolivia | 2 |
| Ireland | 22 | Bosnia and Herzegovina | 6 |
| Sweden | 22 | Botswana | 2 |
| Denmark | 19 | Brazil | 35 |
| Greece | 19 | Brunei | 1 |
| Israel | 19 | Bulgaria | 7 |
| Switzerland | 19 | Burkina Faso | 1 |
| Argentina | 18 | Burundi | 1 |
| Chile | 18 | Cambodia | 2 |
| Malaysia | 18 | Cameroon | 3 |
| Portugal | 18 | Canada | 42 |
| Colombia | 17 | Chile | 18 |
| Indonesia | 17 | China | 43 |
| Philippines | 17 | Colombia | 17 |
| Romania | 17 | Congo | 2 |
| Russia | 17 | Costa Rica | 3 |
| South Africa | 17 | Croatia | 10 |
| Nigeria | 16 | Cuba | 2 |
| Hungary | 14 | Cyprus | 4 |
| Peru | 14 | Czech Republic | 8 |
| Singapore | 14 | Denmark | 19 |
| Bangladesh | 13 | Dominican Republic | 2 |
| Egypt | 12 | Ecuador | 7 |
| Finland | 12 | Egypt | 12 |
| Pakistan | 12 | El Salvador | 3 |
| Saudi Arabia | 12 | Estonia | 7 |
| Serbia | 12 | Ethiopia | 1 |
| Thailand | 12 | Finland | 12 |
| Ukraine | 12 | France | 39 |
| Austria | 11 | Gambia | 1 |
| Hong Kong | 11 | Georgia | 2 |
| Taiwan | 11 | Germany | 78 |
| Vietnam | 11 | Ghana | 4 |
| Croatia | 10 | Greece | 19 |
| Belgium | 9 | Guatemala | 2 |
| Czech Republic | 8 | Honduras | 2 |
| Iran | 8 | Hong Kong | 11 |
| Bulgaria | 7 | Hungary | 14 |
| Ecuador | 7 | Iceland | 3 |
| Estonia | 7 | India | 29 |
| Kazakhstan | 7 | Indonesia | 17 |
| Kenya | 7 | Iran | 8 |
| Morocco | 7 | Iraq | 5 |
| Norway | 7 | Ireland | 22 |
| Slovenia | 7 | Isle of Man | 1 |
| Uruguay | 7 | Israel | 19 |
| Algeria | 6 | Italy | 60 |
| Bosnia and Herzegovina | 6 | Jamaica | 2 |
| Latvia | 6 | Japan | 29 |
| Slovakia | 6 | Jordan | 5 |
| United Arab Emirates | 6 | Kazakhstan | 7 |
| Venezuela | 6 | Kenya | 7 |
| Iraq | 5 | Kuwait | 3 |
| Jordan | 5 | Kyrgyzstan | 1 |
| Cyprus | 4 | Latvia | 6 |
| Ghana | 4 | Lebanon | 4 |
| Lebanon | 4 | Lesotho | 1 |
| Mozambique | 4 | Libya | 2 |
| North Macedonia | 4 | Liechtenstein | 1 |
| Qatar | 4 | Lithuania | 3 |
| Sri Lanka | 4 | Luxembourg | 3 |
| Trinidad and Tobago | 4 | Macao | 1 |
| Tunisia | 4 | Malawi | 1 |
| Uganda | 4 | Malaysia | 18 |
| Afghanistan | 3 | Mali | 2 |
| Azerbaijan | 3 | Mauritius | 2 |
| Belarus | 3 | Mexico | 30 |
| Cameroon | 3 | Mongolia | 2 |
| Costa Rica | 3 | Montenegro | 2 |
| El Salvador | 3 | Morocco | 7 |
| Iceland | 3 | Mozambique | 4 |
| Kuwait | 3 | Myanmar | 2 |
| Lithuania | 3 | Namibia | 2 |
| Luxembourg | 3 | Nepal | 3 |
| Nepal | 3 | Netherlands | 29 |
| Panama | 3 | Nicaragua | 1 |
| Senegal | 3 | Nigeria | 16 |
| Tanzania | 3 | North Macedonia | 4 |
| Albania | 2 | Norway | 7 |
| Angola | 2 | Oman | 1 |
| Armenia | 2 | Pakistan | 12 |
| Bolivia | 2 | Panama | 3 |
| Botswana | 2 | Paraguay | 2 |
| Cambodia | 2 | Peru | 14 |
| Congo | 2 | Philippines | 17 |
| Cuba | 2 | Poland | 35 |
| Dominican Republic | 2 | Portugal | 18 |
| Georgia | 2 | Qatar | 4 |
| Guatemala | 2 | Romania | 17 |
| Honduras | 2 | Russia | 17 |
| Jamaica | 2 | Rwanda | 1 |
| Libya | 2 | Saudi Arabia | 12 |
| Mali | 2 | Senegal | 3 |
| Mauritius | 2 | Serbia | 12 |
| Mongolia | 2 | Sierra Leone | 2 |
| Montenegro | 2 | Singapore | 14 |
| Myanmar | 2 | Slovakia | 6 |
| Namibia | 2 | Slovenia | 7 |
| Paraguay | 2 | Somalia | 1 |
| Sierra Leone | 2 | South Africa | 17 |
| Sudan | 2 | South Korea | 29 |
| Zimbabwe | 2 | Spain | 45 |
| Andorra | 1 | Sri Lanka | 4 |
| Bahrain | 1 | State of Palestine | 1 |
| Barbados | 1 | Sudan | 2 |
| Benin | 1 | Sweden | 22 |
| Brunei | 1 | Switzerland | 19 |
| Burkina Faso | 1 | Syria | 1 |
| Burundi | 1 | Taiwan | 11 |
| Ethiopia | 1 | Tanzania | 3 |
| Gambia | 1 | Thailand | 12 |
| Isle of Man | 1 | Trinidad and Tobago | 4 |
| Kyrgyzstan | 1 | Tunisia | 4 |
| Lesotho | 1 | Turkey | 29 |
| Liechtenstein | 1 | Uganda | 4 |
| Macao | 1 | Ukraine | 12 |
| Malawi | 1 | United Arab Emirates | 6 |
| Nicaragua | 1 | United Kingdom | 109 |
| Oman | 1 | United States | 291 |
| Rwanda | 1 | Uruguay | 7 |
| Somalia | 1 | Uzbekistan | 1 |
| State of Palestine | 1 | Venezuela | 6 |
| Syria | 1 | Vietnam | 11 |
| Uzbekistan | 1 | Yemen | 1 |
| Yemen | 1 | Zambia | 1 |
| Zambia | 1 | Zimbabwe | 2 |

**Tutorial notes for how to use rating system in future evidence evaluations**

The following pages share the verbatim instructions and internal FAQs that were used in carrying out the current review. While those steps should apply broadly, there are some additional factors to consider. These primarily relate to steps that precede sharing the manual, such as selecting topics and evaluating the literature. There may also be additional considerations necessary for some disciplines, or where matters of time are less innate than the current study (i.e., there is little debate that the pandemic began late 2019/early 2020, and the primary reference paper for this study had a set publication date). In these instances, we propose elaborating on the method for future applications and improvements to these protocols.

**Focus of evidence evaluation**

For the current study, the evidence review was dictated directly from 19 claims made in a defined article. This will not always be the case, and the approach can be applied to specific research questions, established conclusions, precise intervention methods or techniques, and treatments. In future iterations, we recommend a much narrower focus, ideally even singular topics. In the case of this evaluation, the 19 claims were based on their original source and their wide impact on broad policy domains. However, this also created complications of uniformity in reviewing, volume of material to review, and even identifying appropriate literature. This can be reasonably avoided in the future by narrowing scope to a smaller number of insights or interventions.

In identifying the target insight(s) or intervention(s), the following points are recommended:

1. Ensure anything to be assessed is written as a testable statement with minimal room for interpretation. Be extremely careful with wording of effects, particularly around directions that only indicate one aspect of an effect (see example).
2. The more specific a statement, the easier it will be to identify relevant literature and limit ambiguity in assessments.
3. Ensure the statement includes precision in effects to the extent being reviewed. For example, is a particular method being assessed for general effectiveness, or only in a specific setting/timeline (e.g., reducing one specific behavior vs any change in behaviors; impact during first six week of the COVID-19 pandemic; impact in elementary school children)?

As an example, here is one approach to avoid, the issue it creates, and one on how to correct it:

Avoid: “Greater levels of exercise are associated with positive emotions.”

Issue: A study finds that greater inactivity is associated with higher levels of depression amongst adults, but does not assess positive emotion nor non-adults. Does it support the initial claim?

Correct: “There is a positive relationship between exercise and positive emotions and a negative relationship between exercise and negative emotions, which are visible across all age groups.”

**Compiling literature**

As with any review, the evaluation team will need to set criteria for the types of literature they will include. Obviously, the nature of rating evidence precludes rules for exclusion based on scale or empirical level, but other criteria must be decided. For example, will you only include peer-reviewed work? Do policy evaluations count? Will you be able to use reports in multiple languages and settings, or are you more concerned about applications in a defined context? How far back will you search, and what is the end point for published articles you will include? Also, what will you do with work that is published during interim between completing searches and finalizing the report?

It is strongly recommended to have a multi-tiered search strategy that involves sophisticated repository engines (e.g., NCBI, PsycINFO) under the guidance of professional library scientists, expert (including academics, users, and practitioners) input, unstructured general search engines, and crowdsourcing. This approach is distinct from a systematic review for practical reasons: it is most important that influential or potentially influential material is reviewed, not only material that would appear in a narrowly defined search strategy. In our study, we had multiple avenues to ensuring significant papers were less likely to be missed, including use of social media to circulate a survey where individuals could submit work. Doing so also helps reduce potential bias of relying on framing used by the research team.

**Data management & extraction**

As papers are identified and formally included in the review, it is highly recommended to extract fundamental details during the compilation. This was extremely useful later in the process, particularly regarding locations, languages, scope, sample sizes, and methods, as well as type of publication (e.g., peer review or policy report). Additional article-specific criteria may be of interest, such as using QUADAS for diagnostic evaluations or TRL for specific products and treatments. We also recommend capturing whether an article is in a pre-print, working paper, first-view, or other non-final format. Those articles can be revisited later to determine if final versions are available. (Note: For this study, we only revisited in some cases because we were interested in what evidence was available by a certain period; that may not always be prudent.)

**Post-evaluation note**

One point that may appear subtle in this manuscript but was ultimately very important is the reconciliation phase of the evaluator ratings. Our process was stated in the methods of our study design prior to the start of evaluations as going directly to the lead investigator to break ties and then presenting those back to the review teams. That approach was done to maintain the anonymity that was central to the approach. In our case, this worked very well due to high agreement in the first round of reviews, so most reconciliation was relatively simple. However, there may be times where agreement is very low, and this information could be reported alongside ratings. Such information will be especially valuable as low agreement may indicate a direct need for more research on a topic or that evidence is being interpreted very differently for a variety of reasons (e.g., ideology, political beliefs, cultural differences, interpretation of cost-benefit).

**MANUAL USED BY ALL REVIEWERS FOR PROJECT**

**Assessing evidence from behavioral science during the COVID-19 pandemic**

**Before starting**

We recommend that you **read through this entire document before beginning** as this will save time and effort, plus hopefully make this a more fun and engaging exercise. To provide quick feedback as questions or concerns arise, we have created an [FAQ S](https://docs.google.com/document/d/1StORlbIReVWJCbGN4vzkKT6qT8ayFAHwn9HU6ByJO2w/edit?usp=sharing)heet [link removed; FAQ pasted later]. Please post your questions there and tag XXX - we will answer them there and others may check (we will occasionally notify the group when this is updated).

To get an idea of the end goal, you may want to review [this report](https://www.gov.uk/government/publications/online-choice-architecture-how-digital-design-can-harm-competition-and-consumers/evidence-review-of-online-choice-architecture-and-consumer-and-competition-harm#oca-practices) (particularly [this section](https://www.gov.uk/government/publications/online-choice-architecture-how-digital-design-can-harm-competition-and-consumers/evidence-review-of-online-choice-architecture-and-consumer-and-competition-harm#oca-practices)). Our work will include more detail in the summary tables, and the overall text will be much shorter, but that report is still a very useful indication.

**Time management**

We have aimed to remove most of the hassle associated with reviewing. To make this an engaging, efficient task, here is an idea of the anticipated workload:

**Part 1:** This is simply to help you locate the core material and take only a few minutes to check. Most critically, it will give you an indication of the volume of reading associated with your claim.

**Part 2:** You should work at a pace you are comfortable with, but it is entirely dependent on the volume of articles for the claim and how many have original, empirical evidence. The assessments for each article are very short (<5 minutes), so you can estimate

**Part 3:** While this is ultimately the most consequential step, it should only take a few minutes to complete this after Part 2 is done. The only point we recommend here is to make sure you have had a chance to reflect on the reading in Part 2 prior to completing it.

**Claims**

**CRITICAL NOTE: Please be certain that you focus on the full text of the claim and *not* the shorthand versions of it. We are assessing the statements made in 2020, so please be sure you are very clear on that text as you assess.**

Articles were allocated to the claims (see final pages) on a cursory review of content. It is very possible that some either are better fit to another claim, or do not belong in the review at all. If this occurs, please notify XXX as early as possible. Evidence should be very closely aligned with the claim statement. There is some flexibility in this in terms of how precisely related they must be, such as emphasizing a single word in a claim beyond its initial intention, but ultimately evidence should only be assessed toward a claim if it appropriately conveys support for or against the statement for policymakers. We provide one example here:

One intervention attempted to mitigate conspiracy beliefs leading to vaccine hesitancy. The claim in the article refers to information *prior to* hearing the conspiracy, but the intervention came after beliefs were identified. We assessed this as appropriate/relevant evidence for the claim anyway.

Another intervention focused on vaccine hesitancy, but focused on matters of fear of needles, (valid) concerns about vaccines, and previous negative experience with healthcare. It did not explicitly look into conspiracy beliefs or misinformation, so it was not included in that claim.

**Procedure**

**Part 1: Open list of assigned articles**

Open the spreadsheet attached to the email with this manual to access the spreadsheet of articles specific to your claim. All articles should have links associated with them for easy access. Contact XXX if any issues accessing an article.

**Part 2 - Reviewing the assigned articles**

For each article, complete the following steps:

1. **Confirm the** **basic details in the spreadsheet** (locked) match your reading of the article, especially if it belongs in a different claim. If any details are wrong, or it does not fit, simply email XXX (no special process, just indicate what seems to be incorrect and what the correct details should be – RAs may assist with this).
   1. If it is a preprint, please just check if there is a fully published version now (for these papers only, we will relax the 31 May cutoff)
2. **Check the article** (RAs may assist with this step)
   1. Does this article belong in **this claim list?** If not, please notify XXX immediately and suggest which claim it should go in.
   2. Does this article directly inform any of the **other claims** from the 2020 paper in a way that it *must* be considered in those reviews as well? You can find a list of all claims at the end of this document.
   3. It may be acceptable – or even advisable – to **skip the introductions**, but please at least ensure there is nothing directly relevant to your final assessment in those sections. Also do not be swayed by claims in the title, abstract, or introduction – we are assessing evidence, not superlatives.
   4. If your folder has many articles (e.g., >15), **you may also skip any articles that clearly do not have new empirical evidence and simply rate them as 1**. It is also fine to review and include notes for these, but that is primarily valuable when there are few papers overall.
3. **What you will add** in the spreadsheet *(repeat for each article)*:
   1. What is the **overall direction** of the finding related to the claim (positive, negative, null, mixed - or N/A due to not being an empirical study)?
      1. For assistance on determining your response, answer this question: “If this approach were implemented as an intervention, based on the evidence available, what would the expected direction be?” This can also be addressed in the summary statement.
      2. Please try to limit use of “mixed” to only when there are truly conflicting directions from the highest levels of evidence available (it is likely to always be mixed in some ways, but we really want to know the direction of the best/highest evidence).
   2. If there is an **overall effect**, is it small, medium, or large?
      1. Reviewers are free to determine their own thresholds and clarify in summary if necessary. “Null” should only be used for empirical study with no effect. If no empirical evidence exists, leave blank and simply give the 1 rating for the overall assessment.
   3. What is your **overall assessment** of the evidence level of the article related to the claim (whether for or against)?
      1. 1 - It is only a review or an opinion; there is only theory, **not empirical** evidence
      2. 2 - There is **some empirical evidence** relevant to the claim, but it is not from real-world contexts (e.g., only from controlled lab environments or surveys)
      3. 3 - There is a **clear application relevant to the claim** with empirical, real-world/consequential study
      4. 4 - There is **evidence of replication of effects of application** in real-world settings
      5. 5 - There is **large-scale evidence of real-world impact** relevant to the claim
      6. Note 1: Only pick **one overall value** - no partials or multi-rating. If a single article has multiple arms, we will request separate reports for each, if necessary (if they all come to the same agreement, this may not be required)
      7. Note 2: Any work that has not been carried out in real-world settings cannot receive above a 2 rating. Surveys can count as real-world so long as true behavior is captured. Contained experiments, even if replicated multiple times in multiple languages/countries, only receive a 2 if there is no real-world validation.
      8. Note 3: **What if the study is poor quality?** This is very likely and there are three options:
         1. Simply don’t rate it. Put a note that it is too poor to consider as evidence.
         2. Rate it at the level it should be rated at, but note that you do not plan to consider it in the full claim assessment
         3. Give a downgraded rating. For example, a poor quality, real-world trial could be treated as 2 stars (i.e., there is evidence, but not at the level it superficially appears to be).
   4. Any **other notes** or concerns - keep these brief; they are not mandatory. **Maximum 100 characters** per article.

**Part 3 - Reporting your assessments and rating the overall claim**

Only **after completing all article reviews**, go to the second tab and complete the following:

1. In your view, which **assessment** value is most applicable, based on the evidence reviewed relevant specifically to the original claim? Just pick one of these from the dropdown.
   1. **1 - Theory** (there is no empirical work to either support or negate this claim)
   2. **2 - Empirical** (there is some evidence, but minimal or limited to controlled settings, surveys)
   3. **3 - Applicable** (there is sufficient real-world evidence of an effect, though not widely generalizable or limited to a very specific setting)
   4. **4 – Replication*** (there is *converging* evidence of *application* that has been found in multiple contexts; if replications have been unsuccessful, revert to 3)
   5. **5 – Impact*** (concept has been validated in real-world at scale; note that this does not mean 100% success rate and in fact, may be more useful with ‘contours’ as opposed to universality - does not require multiple studies at scale, but the highest level one must be extremely strong if only one exists)
   6. Note 1: You are providing the rating for the *highest level of evidence*, not an average across all (i.e., even if 19 out of 20 articles are not empirical, if the 20th article is a global study with real world effects observed, you can give it a 5 if you feel it is appropriate). This is entirely left to your assessment. If you feel the largest-scale studies are of low quality and assess a lower result, this is also acceptable.
   7. Note 2: Only use whole numbers and a single score. This may be frustrating, but just recall that others are rating as well, so you are not the only rating being provided.
   8. Note 3: “Replication” only applies to findings in real-world settings. Replications of lab experiments in controlled settings should only receive a 2.
   9. *A rating of 4 or 5 for an overall evidence base without a single study alone appearing as a 4 or 5. This is what separates 2s from 3-4-5. A large number of high-quality 3s and 4s could collectively mean the overall evidence base is very high, even if no single study testing everything all at once. This is a fundamental difference between evidence for policy and simply scientific evidence. A collection of individual studies can mean an overall value greater than their value in isolation.
2. **Summarize the rating** (maximum 200 characters)
   1. Note: It is *your* rating. You do not have to justify it. Only summarize the basis.
3. Report an **overall direction** (positive, negative, null, no evidence)
   1. Note: Results are likely to be heterogeneous and therefore “mixed” will often be the preferred response. We ask you to avoid this except where absolutely unavoidable. Again, this is your summary of *the best evidence available*, not a summary of all findings. “Mixed” would only truly apply if multiple studies of equal quality and rigor produce opposing results.
4. Report an **overall effect size** (large, medium, small, null, no evidence available)
   1. Note: This only relates to the best evidence available (whether one study or ten).
   2. For assistance on determining your response, answer this question: “If this approach were implemented as an intervention, based on the evidence available, what would the expected effect size be?” This can also be addressed in the summary statement.
5. Provide the archetype or ‘best’ study/article (ideally not your own work but not prohibited!) that you feel is most indicative or supportive of your final rating for ‘best evidence’

**When you have completed reviewing articles and filling in the second tab in the spreadsheet, please simply click here [email link removed] and return the completed spreadsheet to XXX.**

**Claims**

**Statements in bold come from Box 1 in the original paper.** Statements in plain text were not in Box 1 but did come directly from the article. *Statements in italics have been modified from the original text for the purposes of assessment.*

1. **A shared sense of identity or purpose can be encouraged by addressing the public in collective terms and by urging ‘us’ to act for the common good.**
2. **Identifying *trusted* sources (for example, *local*, religious, or community leaders) that are credible to different audiences to share public health messages can be effective.**
3. **Leaders and the media might try to promote cooperative behavior by emphasizing that cooperating is the right thing to do and that other people are already cooperating.**
4. **Norms of prosocial behavior are more effective when coupled with the expectation of social approval and modeled by in-group members who are central in social networks.**
5. **Leaders and members of the media should highlight bipartisan support for COVID-related measures, when they exist, as such endorsements in other contexts have reduced polarization and led to less-biased reasoning.**
6. **There is a need for more targeted public health information within marginalized communities ~~and for partnerships between public health authorities and trusted organizations that are internal to these communities.~~ [We will mostly ignore the second half of this statement as it relates more to managerial practices than behavioral science.]**
7. **Messages that (i) emphasize benefits to the recipient, (ii) focus on protecting others, (iii) align with the recipient’s moral values, (iv) appeal to social consensus or scientific norms and/or (v) highlight the prospect of social group approval tend to be persuasive. [This statement may need to be separated for the final assessment]**
8. **Given the importance of slowing infections, it may be helpful to make people aware that they benefit from others’ access to preventative measures.**
9. **Preparing people for misinformation and ensuring they have accurate information and counterarguments against false information before they encounter conspiracy theories, fake news, or other forms of misinformation, can help inoculate them against false information.**
10. **Use of the term ‘social distancing’ might imply that one needs to cut off meaningful interactions. A preferable term is ‘physical distancing’, because it allows for the fact that social connection is possible even when people are physically separated.**
11. As negative emotions increase, people may rely on negative information about COVID-19 more than other information to make decisions. In the case of strong emotional reactions, people may also ignore important numeric information such as probabilities and a problem’s scope.
12. Cultures accustomed to prioritizing freedom over security may also have more difficulty coordinating in the face of a pandemic.
13. *Fake news, conspiracy theories, and misinformation will have a negative impact on vaccine hesitancy.*
14. *Unmitigated political polarization will disrupt or create other negative effects on attempts to minimize or end the pandemic.*
15. *Active use of online connections can reduce some negative mental and other health effects created by isolation policies. (Note: Examples of the harm created by passive use of social media may actually count as supportive evidence of this statement)*

**FAQ for behavioral policy evaluation report [Material provided to and updated for all reviewers during process]**

**Please write your questions below in red** and tag XXX by putting “@[email address removed]” at the end, which will let me know it is there. Answers will be provided as quickly as possible. **Please just keep all questions anonymous!** (Email me if you want a notification when I have answered.)

**How do I fix the ‘effect size’ cell when I do the full evaluation?**

There is a minor flaw in the ‘effect size’ cell when you go to the second tab. There are four ways to fix this (you only need to do one):

1. **PREFERRED**: Click on the cell. Go to Data > Data Validation (under Data Tools). Change “B” to “C” in the “Source” line (twice) - or simply copy/paste this exactly:
   - - - 1. ='Dropdown lists'!$C$1:$C$4
2. Ask XXX for a corrected version.
3. State the effect size (small, medium, or large) in the summary statement (KR will add in).
4. Add a comment over the cell to say if it should say small, medium, or large.

**2. What about a high quality study that we don’t feel actually speaks to the claim?**

Evaluate the article as you feel it deserves to be evaluated, and then either leave the rating blank, or rate it as it should be but do not include in your final rating (suggesting making a note about this at the end). It is really not surprising to find quite a few like this. **You may ignore the manual where it says to notify XXX about irrelevant papers - just put in the notes as we can track it that way.**

**3. What about lots of papers that don’t directly assess the claim?**

As in the previous one, you may either skip these entirely (may help to make notes) or assess them as they are, but you may disregard them when it comes to your final rating of the overall claim. **You may ignore the manual where it says to notify XXX about irrelevant papers - just put in the notes as we can track it that way.**

**4. The manual says “if it is a preprint, please just check if there is a fully published version now”. What shall we do if there is a fully published version? Notify XXX?**

Yes, in this case: please review the published article rather than the preprint and notify me of the switch. I will make this note at the end of this FAQ so we can ensure everyone has done this.

**5. What about self-report in the real world?**

Assuming it is good quality research and has no other limitations, you should assess this at the level you feel it works. For example, if these are clinical evaluations, then 3 would certainly fit. However, if you feel they aren’t strong enough, it is okay to treat as 2. Alternatively, if you feel the quality is fine and technically it meets a 3, but you don’t feel it actually advanced the research, rate it as a 3 but do not factor it in to your overall assessment. Suggest making a note of this in the final column.

**6. What about a lot of 3s from different locations?**

It is very likely that you will find a lot of decent evidence that aren’t specifically from articles treated as replications or testing in a number of settings. This is the major reason why there is only one broad category for limited empirical evidence, and essentially three levels for real-world testing. Many 3s with converging evidence on a topic can become a 4 or a 5, unlike 2s - many 2s are nice, but they do not validate impacts, so they cannot become a 3.

**7. What if the study is poor quality?**

This is very likely and there are three options:

1. Simply don’t rate it. Put a note that it is too poor to consider as evidence.
2. Rate it at the level it should be rated at, but note that you do not plan to consider it in the full claim assessment
3. Give a downgraded rating. For example, a poor quality, real-world trial could be treated as 2 stars (i.e., there is evidence, but not at the level it superficially appears to be).

**8. Why are we allowing preprints and what are we meant to do with them?**

For better or for worse, preprints had a major impact on policy during the pandmeic. You are not required to incorporate your ratings of these into your final/overall assessment. However, given their influence, we need to incorporate these into our overall review. If you think they are not good enough, you may leave the evidence rating empty. We are not forcing anyone to include anything (preprint or otherwise), but we do want to assess the volume of relevant preprints and present some sort of indication of their suitability to inform policy (beyond simply “not all preprints are alike”).

**9. If my claim is about a negative impact of x on y, and papers find this result, does this count as “negative” or “positive” in the direction of effects?**

Positive. In this case, we’re only determining whether the effect supports the claim, so it is a positive effect, as confusing as this may be. However, remember that we defer to the reviewer’s assessment. If you determine the work isn’t as directly linked to the claim, then you do not need to leave a rating.

**10. If we find additional papers relevant to the claim, is there any chance they can still be added?**

We definitely can review additional papers, but the guidelines will be:

1. They still have to meet all the criteria, particularly that they were published prior to/on May 31, 2022.
2. They should be impact the final rating (there is no point to review an article that has no impact on the final assessment, though we can at least produce a list of ‘stuff we found after we started’)
3. You do *not* need to send these to XXX yet, please just keep a list and we will make a determination later about augmenting any assessments.

**11. What about literature review papers? Should they be included?**

*Narrative* literature reviews are almost automatically a 1 - no need to go further, though completely fine to make a note if there’s a relevant thing to know about the review.

*Systematic* reviews can potentially count, but the idea would need to be that they are using articles we are reviewing independently. There’s more discretion left to you as reviewers how to make use of those. If you feel they synthesize in a meaningful way, then they certainly can be useful as 4s and 5s. However, if they only synthesize a lot of 2s, then they don’t really add anything (assuming we have the original papers already in the list).

**12. What if we cannot access the full article because of a paywall?**

Notify XXX immediately and I will provide - if I cannot and also cannot get a copy from the authors, we will skip it.

**13. The overall direction is missing the N/A option for overall direction in the sheet. However, the guide mentions that this option should be used for non empirical studies. How should we proceed for non empirical studies - leave blank?**

From the manual: *If no empirical evidence exists, leave blank and simply give the 1 rating for the overall assessment.*

**14. Claim 4 (social identity) states “Norms of prosocial behavior are more effective when coupled with the expectation of social approval and modeled by in-group members who are central in social networks.” So, does this mean that we only include studies that directly compare e.g. norms coupled with social approval vs. without social approval or norms with regards to friends vs. people in general? And if we have an article that states, for example, that social norms are associated with higher compliance behavior, would that article be even relevant to the claim?**

This is another of the tricky ones in that it is really down to how each reviewer reads the claim. It would make sense to focus your final assessment on articles that very specifically meet all aspects of the statement, but I wouldn’t necessarily ignore the other ones as it may be extremely helpful to know (in the final assessment) if many studies touched on certain aspects but not all, and if the read of evidence needs to be more nuanced because of it (e.g., one aspect is strongly supported; another not).

**15. I have a follow up to Q14. I am re-reading the original paper to try and distinguish Claims 3 and 4. Is claim 3 looking at general evidence supporting the role of interventions using descriptive norms (perceptions of what others do) or injunctive / prescriptive norms (messages highlighting approval of behaviors)? And claim 4 looking at if norms interventions work better if norms are provided / modeled by an in-group member (moderation hypotheses) or if both descriptive and injunctive are included? I wonder if some of the papers assigned to claim 4 are also relevant to claim 3?**

Certainly many articles are relevant to multiple claims, and you are certainly encouraged to keep a list of any that you think absolutely must be considered in other claims. However, knowing that this could end up with hundreds of papers in each claim, we have mostly suggested recommending only those that you feel would likely move the needle on the overall assessment (i.e., only propose articles that seem to be fundamentally critical for assessing other claims). We will call for those later on in a ‘post-mortem’ after assessments are done. We did include a few articles in multiple claims where it was especially apparent that they applied in both, but we tried to limit these for the reasons listed here.

**16. The manual states: ‘If a single article has multiple arms, we will request separate reports for each, if necessary’. How do I report that without changing the formatting of the spreadsheet?**

Please just notify me when you submit your reviews of this aspect. I will modify the one you send back and add in those details for you so long as they are clear in the email. It is up to you if you want to incorporate this into the overall assessment.

**NOTES FOR SPECIFIC CLAIMS**

**Claim 3**

For Kukowski et al. (2021) - add behavior: social distancing

**Claim 11**

There are two related claims here. Do we choose which one we rate for direction, effect, and assessment? Or are articles only relevant if they include data relevant to both claims and then we rate based on some combination across the two claims?

The best approach here would be to rate the articles in line with whichever aspect of the claim they fit. However, when you go to write the overall assessment, you could itemize the summary statement. E.g. “For the first part, there was wxyz quality/depth/study types/impacts. For the second, it was more abcd.” If they end up being very similar, you can be brief, but if they require very different summaries, then I’m happy to discuss modifying the reporting mechanism to allow two distinct rows. I will send a brief note to all reviewers on claim 11.

**Claim 12**

Which particular cultural dimensions do we count as pertaining to the freedom/security claim? E.g., are individualism/collectivism, tightness/looseness, and relational mobility all equally relevant?

This is 100% up to the reviewers. I would not want to limit or constrain these assessments in any way. I would only suggest putting this in your notes for articles and refer to it in the final assessment if relevant (e.g., if certain ones are heavily used or completely ignored).

How can I select a different article from the updated list on the second tab?

Click on the cell. Go to Data > Data Validation (under Data Tools). Change “12” to “35” in the “Source” line - or simply copy/paste this exactly:

=Articles!$C$2:$C$35

**Claim 13**

**Fake news, conspiracy theories, and misinformation will have a negative impact on vaccine hesitancy.**

Is this supposed to mean “negative impact” in the sense of it is “bad” because of increasing vaccine hesitancy or is this supposed to mean “negative impact” in the sense of a “negative relationship”, i.e. decreasing vaccine hesitancy? (I’ll assume the former for now but want to make sure I get the overall direction etc right and may be worth rewording in either case? e.g. negative impact on vaccine acceptance)

I think the most sensible read is that “negative” would imply hesitancy based on factors other than facts, health (individual or public), or genuine concerns. So the direction implies that more of those patterns (fake news, etc.) will lead to more people *not* getting vaccinated.

**Claim 14**

Gollwitzer et al is not a preprint, but peer review.

**Claim 15**

Bentley et al (2021) is an uncontrolled trial, not a survey.

Claim 15 clarification - Active use of *online connections* can reduce mental health effects… - does this include only social online connections? Or does it cover use of online media with no interaction (eg reading news).

Reading news would not count as an online *connection*, so anything that is simply reading without interaction would not qualify (not even as passive use - because it is entirely about social connections, not just reading information; passive use would be scrolling/occasionally liking or posting, but not actually engaging in interaction). This should be noted in your assessment of the claim if it is a common feature in the studies in your list. If it involved something like reading news plus posting in comments, that would likely count as passive, but I would also note this in the final assessment.
