# Supplementary material for: A synthesis of evidence for policy from behavioural science during COVID-19
Source: Nature. 2023 Dec 13;625(7993):134–47. doi: 10.1038/s41586-023-06840-9 (PMC10764287; doi:10.1038/s41586-023-06840-9)
Supplement: Supplementary file 2 — Reporting Summary [file 41586_2023_6840_MOESM2_ESM.pdf]

nature portfolio

Corresponding author(s): Kai Ruggeri

Last updated by author(s): Sep 29, 2023

Reporting Summary

Nature Portfolio wishes to improve the reproducibility of the work that we publish. This form provides structure for consistency and transparency in reporting. For further information on Nature Portfolio policies, see our Editorial Policies and the Editorial Policy Checklist.

Statistics

For all statistical analyses, confirm that the following items are present in the figure legend, table legend, main text, or Methods section.

n/a	Confirmed	
☒	☐	The exact sample size (*n*) for each experimental group/condition, given as a discrete number and unit of measurement
☒	☐	A statement on whether measurements were taken from distinct samples or whether the same sample was measured repeatedly
☒	☐	The statistical test(s) used AND whether they are one- or two-sided<br>*Only common tests should be described solely by name; describe more complex techniques in the Methods section.*
☒	☐	A description of all covariates tested
☒	☐	A description of any assumptions or corrections, such as tests of normality and adjustment for multiple comparisons
☒	☐	A full description of the statistical parameters including central tendency (e.g. means) or other basic estimates (e.g. regression coefficient) AND variation (e.g. standard deviation) or associated estimates of uncertainty (e.g. confidence intervals)
☒	☐	For null hypothesis testing, the test statistic (e.g. *F*, *t*, *r*) with confidence intervals, effect sizes, degrees of freedom and *P* value noted<br>*Give P values as exact values whenever suitable.*
☒	☐	For Bayesian analysis, information on the choice of priors and Markov chain Monte Carlo settings
☒	☐	For hierarchical and complex designs, identification of the appropriate level for tests and full reporting of outcomes
☒	☐	Estimates of effect sizes (e.g. Cohen's *d*, Pearson's *r*), indicating how they were calculated

Our web collection on statistics for biologists contains articles on many of the points above.

Software and code

Policy information about availability of computer code

Data collection	All data utilized in this study came from qualitative summaries of detailed reviews of published evidence. Studies were compiled using online search engines (Google Scholar, NCBI, PsycINFO, EconLit), crowdsourcing on social media (primarily Twitter and LinkedIn), and through a Qualtrics upload document. Study information were then stored on a shared spreadsheet (Google Sheets) before downloading to Excel. Interactive data were eventually uploaded on the Tableau Public software platform. Some methods details were extracted from each study, such as the country or countries where the study took place, sample size, general method, and source of publication.
Data analysis	The goal of this paper was to synthesize evidence, therefore the primary methods involved to analyze claims are described qualitatively. No inferential statistical analyses are presented in the main text. The only quantitative analyses are presentation of raw numbers, typically divided by 19 (the number of claims assessed). A small number of mean ratings are also produced but no NHST is presented with them. There are two mentions of t-tests in supplemental information, but these are only for indications of internal consistency and rely on small-n analyses.

For manuscripts utilizing custom algorithms or software that are central to the research but not yet described in published literature, software must be made available to editors and reviewers. We strongly encourage code deposition in a community repository (e.g. GitHub). See the Nature Portfolio guidelines for submitting code & software for further information.

Data

Policy information about availability of data

All manuscripts must include a data availability statement. This statement should provide the following information, where applicable:

- Accession codes, unique identifiers, or web links for publicly available datasets
- A description of any restrictions on data availability
- For clinical datasets or third party data, please ensure that the statement adheres to our policy

All data and study material is provided either in the supplementary information or through the two online repositories (OSF and Tableau Public, both accessible via https://psyarxiv.com/58udn). The precise OSF link will be provided with the final publication (we will upload all material once there are final versions of the manuscript and supplement). It will also be accessible via the current page (https://psyarxiv.com/58udn), where the pre-print and interactive tool are already available.

Human research participants

Policy information about studies involving human research participants and Sex and Gender in Research.

Reporting on sex and gender	Not applicable
Population characteristics	Not applicable
Recruitment	Not applicable
Ethics oversight	Not applicable

Note that full information on the approval of the study protocol must also be provided in the manuscript.

Field-specific reporting

Please select the one below that is the best fit for your research. If you are not sure, read the appropriate sections before making your selection.

☐ Life sciences ☒ Behavioural & social sciences ☐ Ecological, evolutionary & environmental sciences

For a reference copy of the document with all sections, see nature.com/documents/nr-reporting-summary-flat.pdf

Behavioural & social sciences study design

All studies must disclose on these points even when the disclosure is negative.

Study description	This study involved compiling and assessing all publications related to behavioral science during the pandemic to inform scientists and policymakers about the accuracy of expectations made in April 2020. Studies were then rated based on the level of evidence they provided, ranging from pure theory/opinion to large-scale, real-world impact.
Research sample	All data used involved publicly available scientific materials that related to or directly tested claims made the Van Bavel et al., 2020 article. No human participants were involved. All sampling involved reviewing studies published from late 2019 through May 2022. A final repository of 747 articles was used for the analyses in this study.
Sampling strategy	Articles and reports were identified and considered for the assessment through extensive systematic and manual searches by all evaluators, with the primary criterion being that they were publicly available prior to June 1, 2022. Searches included using the systematic review criteria produced by PubMED-NCBI for research specifically on COVID-19, as well as checking preprint servers, multiple repository search engines, crowdsourcing (on social media and targeted email lists), and snowballing of relevant articles (including articles that cited the original paper). There was no restriction for locations or language (see later for a discussion of the ways in which diversity of authorship enabled broader searching).
Data collection	All data utilized in this study came from qualitative summaries of detailed reviews of published evidence. Studies were compiled using online search engines (Google Scholar, NCBI, PsycINFO, EconLit), crowdsourcing on social media (primarily Twitter and LinkedIn), and through a Qualtrics upload document. Study information were then stored on a shared spreadsheet (Google Sheets) before downloading to Excel. Interactive data were eventually uploaded on the Tableau Public software platform.
Timing	The search for articles begin formally in February 2022 and was stopped on May 31, 2022. Some papers that met the full criteria were identified through snowballing or brought to the attention of the research team through other means (e.g., news reports, social media posts, papers identified in the review of studies included originally). However, studies were only permitted to be added after the 1st of June, 2022, if they met all other inclusion criteria. This approach was taken to ensure we minimized the possibility of missed studies due to the short turnaround for search engines to identify papers produced in a generally narrow timeline.

Data exclusions	No studies published on/after June 1, 2022, were permitted. Studies published in late 2019 were permitted, provided that they specifically focused on COVID-19. Some studies were published as pre-prints prior to June 1, 2022, but were later identified as having been published through peer-review after the deadline. When this was identified, reviewers were encouraged to use the peer-reviewed version as it was presumed to be more reliable evidence.
Non-participation	Not applicable.
Randomization	Not applicable.

Reporting for specific materials, systems and methods

We require information from authors about some types of materials, experimental systems and methods used in many studies. Here, indicate whether each material, system or method listed is relevant to your study. If you are not sure if a list item applies to your research, read the appropriate section before selecting a response.

Materials & experimental systems

n/a	Involved in the study
☒	☐ Antibodies
☒	☐ Eukaryotic cell lines
☒	☐ Palaeontology and archaeology
☒	☐ Animals and other organisms
☒	☐ Clinical data
☒	☐ Dual use research of concern

Methods

n/a	Involved in the study
☒	☐ ChIP-seq
☒	☐ Flow cytometry
☒	☐ MRI-based neuroimaging
